# PROteolysis‐Targeting Chimeras (PROTACs) in leukemia: overview and future perspectives

**DOI:** 10.1002/mco2.575

**Published:** 2024-06-05

**Authors:** André T. S. Vicente, Jorge A. R. Salvador

**Affiliations:** ^1^ Laboratory of Pharmaceutical Chemistry Faculty of Pharmacy University of Coimbra Coimbra Portugal; ^2^ Center for Neuroscience and Cell Biology University of Coimbra Coimbra Portugal; ^3^ Center for Innovative Biomedicine and Biotechnology (CIBB) University of Coimbra Coimbra Portugal

**Keywords:** bifunctional molecule, degrader, hematological malignancy, leukemia, PROteolysis‐TArgeting Chimera (PROTAC), targeted protein degradation

## Abstract

Leukemia is a heterogeneous group of life‐threatening malignant disorders of the hematopoietic system. Immunotherapy, radiotherapy, stem cell transplantation, targeted therapy, and chemotherapy are among the approved leukemia treatments. Unfortunately, therapeutic resistance, side effects, relapses, and long‐term sequelae occur in a significant proportion of patients and severely compromise the treatment efficacy. The development of novel approaches to improve outcomes is therefore an unmet need. Recently, novel leukemia drug discovery strategies, including targeted protein degradation, have shown potential to advance the field of personalized medicine for leukemia patients. Specifically, PROteolysis‐TArgeting Chimeras (PROTACs) are revolutionary compounds that allow the selective degradation of a protein by the ubiquitin–proteasome system. Developed against a wide range of cancer targets, they show promising potential in overcoming many of the drawbacks associated with conventional therapies. Following the exponential growth of antileukemic PROTACs, this article reviews PROTAC‐mediated degradation of leukemia‐associated targets. Chemical structures, in vitro and in vivo activities, pharmacokinetics, pharmacodynamics, and clinical trials of PROTACs are critically discussed. Furthermore, advantages, challenges, and future perspectives of PROTACs in leukemia are covered, in order to understand the potential that these novel compounds may have as future drugs for leukemia treatment.

## Graphical abstract

Antileukemic PROteolysis‐TArgeting Chimeras (PROTACs) induce degradation of leukemia‐associated targets, including resistance‐conferring mutant forms. PROTACs against leukemia have increased dramatically. Most antileukemic PROTACs, through an event‐driven mechanism, degrade the target potently, quickly, selectively, and with a long duration of action in vitro and in vivo, overcoming the difficulties of conventional inhibitors. Currently, six antileukemic PROTACs are in phase I/II of clinical trials

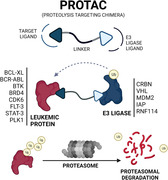

## 1 INTRODUCTION

Among the several main culprits threatening human health, cancer has been considered as one of them since the 20th century, and it remains a threat to this day, with data from GLOBOCAN estimating a total of 16.3 million deaths from cancer between 2020 and 2040.^1^, ^2^, ^3^ Of these, 479,000 will be due to leukemia, a hematological cancer caused by errors in differentiation, growth, and apoptosis of hematopoietic stem cells (HSCs) in either lymphoid or myeloid lineages.^4^, ^5^, ^6^

Hematopoiesis is an extremely dynamic and complex lifelong process of continuous formation and renewal of blood cells.^7^, ^8^ During this process, pluripotent HSCs give rise to progenitor cells capable of differentiating into lymphoid or myeloid lineages, giving rise to all specialized blood cells.^9^, ^10^ However, exposure to ionizing radiation, alkylating agents, viral infections, among other factors not yet identified, can trigger damage to HSCs, resulting in the loss of their ability to activate the cellular repair mechanism, thus transforming them into abnormal leukocytes, called leukemic cells, with genetic errors, altered functions, and increased proliferative capacity.^11^, ^12^ Thus, these leukemic cells end up entering a process of clonal expansion, replacing and disrupting the normal physiology of blood cells, with the triggering of clinical symptoms and signs, which are eventually diagnosed as leukemia.^13^, ^14^

**FIGURE 1:**
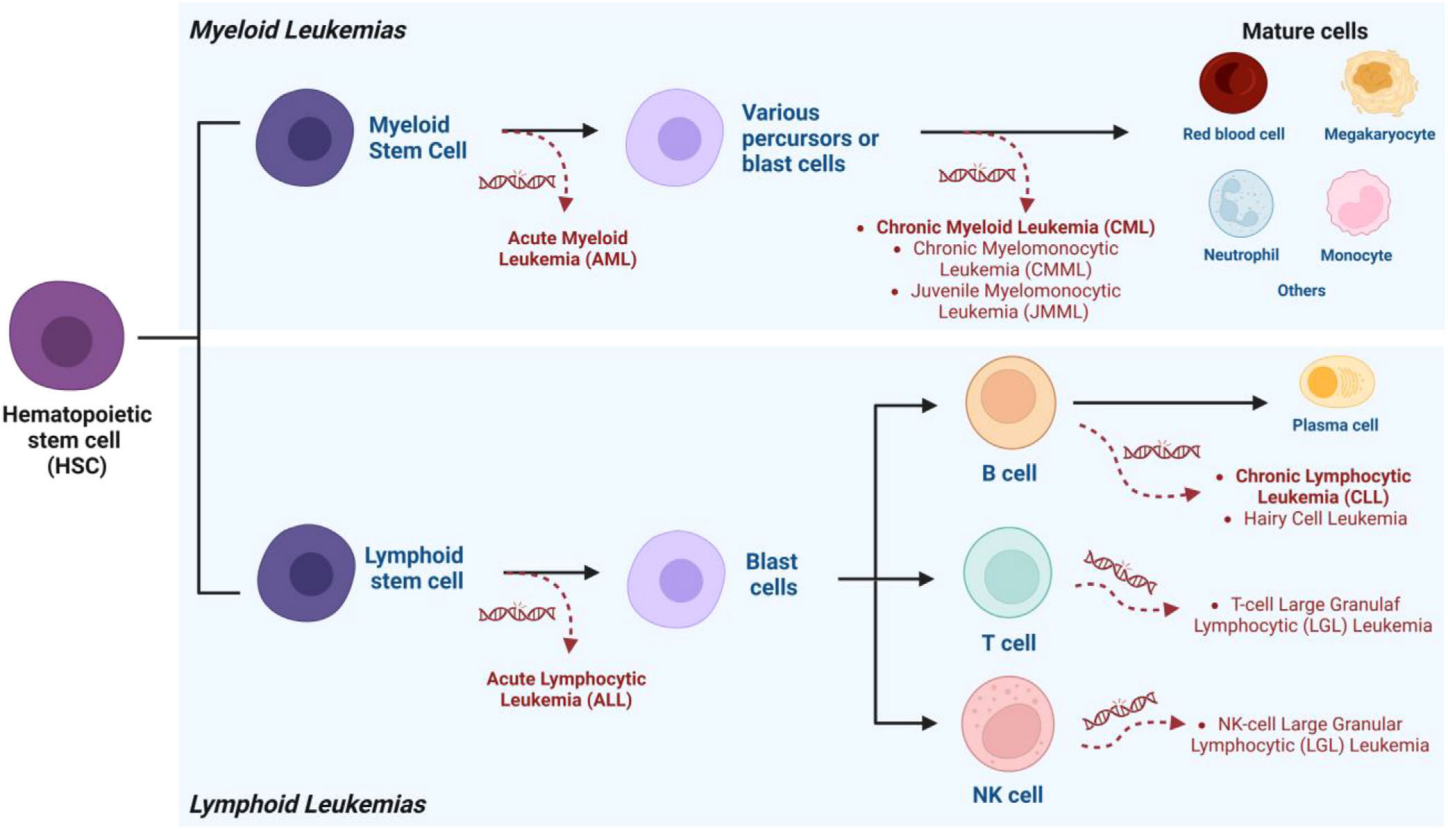
Hematopoiesis and the origin of different types of leukemia. Throughout the complex process of hematopoiesis, exposure to ionizing radiation, alkylating agents, viral infections, and other as yet unidentified factors can damage hematopoietic cells, transforming them into leukemic cells and triggering the clinical symptoms and signs diagnosed as leukemia. Leukemia is a heterogeneous group of life‐threatening malignancies, and its various types can be divided into two groups based on whether they originate from lymphoid or myeloid cells.^9^, ^11^, ^12^

Leukemia is a heterogeneous group of life‐threatening malignant disorders resulting from the dysfunctional proliferation of abnormal leukemic cells that develop in the bone marrow and blood.^15^, ^16^ Simplistically, there are different subtypes of leukemia, broadly classified in two ways. First, according to the severity of the symptoms, such as acute (with severe to very severe symptoms, associated with rapid development) or chronic (with mild to moderate symptoms, associated with slow development).^17^, ^18^, ^19^ Lymphocytic (also known as lymphoid or lymphoblastic) leukemia starts in lymphoid cells, while myeloid (also known as myelogenous) leukemia starts in myeloid cells.^16^, ^20^, ^21^ Therefore, the four main types of leukemia are acute lymphocytic leukemia (ALL), acute myeloid leukemia (AML), chronic myeloid leukemia (CML), and chronic lymphocytic leukemia (CLL) (Table 1).^22^, ^23^, ^24^, ^25^, ^26^, ^27^, ^28^, ^29^, ^30^ In addition to these types of leukemia, there are others that are less common, as illustrated in Figure 1.^16^

**TABLE 1:** The most common types of leukemia.

Leukemia type		Information	References
Acute lymphocytic leukemia	ALL	Is a rare and rapidly progressing leukemia, which without treatment can be fatal within a few months. It is the most common type in the pediatric population (80% of cases), but it can also occur in adults (20% of cases). ALL can be divided depending on the lymphoblast of origin into B or T‐cell variants.	22, 23
Acute myeloid leukemia	AML	Accounts for more than 80% of all cases of leukemia in adults, and around 25% in children; however, it represents less than 1% of all cancers. It is the most aggressive type, and given its complexity, its prognosis depends on its molecular subtype.	24, 25
Chronic myeloid leukemia	CML	Corresponds to more than 15% of all diagnosed cases of leukemia and is usually treatable. It is common in people over 65 years of age, with slow growth, and asymptomatic during the initial months or years.	26, 27
Chronic lymphocytic leukemia	CLL	Accounts for around 25% of all cases of leukemia, frequently diagnosed in older adults, and without the need to initiate treatment until symptoms worsen.	28, 29

Although treatment depends on the type of leukemia, the most common strategy is based on immunotherapy, chemotherapy, or stem cell transplant.^22^, ^24^, ^26^, ^28^ Chemotherapy, through the use of small molecule inhibitors (SMIs), has shown remarkable results.^31^ However, the occurrence of serious side effects, long‐term sequelae, relapses, development of resistance, lifelong use, among others, constitute major concerns, which require the search for innovative, safer, and more effective therapeutic alternatives.^6^, ^32^ In order to overcome these difficulties, investigations have essentially focused on the development of new inhibitors, the research and study of new therapeutic targets, as well as the development of new therapeutic strategies. Among the novel therapeutic strategies that have emerged over the last few years, targeted protein degradation using bifunctional molecules known as PROteolysis‐TArgeting Chimeras (PROTACs) has demonstrated unprecedented results.^33^, ^34^, ^35^

PROTACs have been shown to be able to overcome some of the disadvantages of conventional therapies, with very promising results, both in vitro and in vivo.^36^, ^37^, ^38^ In such a way that they have progressed through various stages of preclinical and clinical development for the treatment of several types of diseases, with a special focus on cancer treatment.^39^, ^40^ Consequently, the study of its therapeutic potential in a wide range of diseases, including leukemia, has been very extensive.^41^ Some antileukemic PROTACs have shown excellent activity against relevant targets involved in the development of one or more types of leukemia. Some of them are already in clinical trials and have demonstrated that they can overcome some of the major drawbacks associated with the use of SMIs, such as the emergence of resistance, thus enabling a leap away from the current inhibitor‐based drug paradigm.

Given the sharp increase in the number of antileukemic PROTACs in recent years, it is essential to analyze and discuss the available information in order to build new insights for future research. Therefore, the present review summarizes the PROTACs intended for the treatment of one or more distinct types of leukemia by degrading key leukemia‐associated targets, presented here by therapeutic target and chronological order. Furthermore, it also aims at a critical analysis and discussion accompanied by future perspectives on antileukemic PROTACs. All articles were analyzed, and the most representative pharmacological PROTACs presented therein are described mainly in terms of chemical structure, degradational and pharmacological activities, structure–activity relationship (SAR) studies, and future potential clinical applications.

## 2 OVERVIEW OF PROTACs

Although it has been more than 20 years since the first PROTAC was reported by the Crews’ laboratory in 2001, their development has exploded in the last 5 years, with more than 3270 PROTACs now reported according to the PROTAC‐database (PROTAC‐DB) (http://cadd.zju.edu.cn/protacdb/).^42^, ^43^, ^44^, ^45^

PROTACs are bifunctional molecules that induce a selective posttranslational degradation of a protein of interest (POI) using the cell's own machinery, by the ubiquitin–proteasome system (UPS).^46^, ^47^ The UPS is one of the major intracellular protein degradation pathways, in which proteins are first poly‐ubiquitinated and subsequently eliminated by the 26S proteasome.^48^ In more detail, the globular protein ubiquitin is activated by the ubiquitin‐activating enzyme E1, in an ATP‐dependent process, and is then transferred to the ubiquitin‐conjugating enzyme E2.^49^ The ubiquitin–E2 enzyme complex binds to the E3 ubiquitin ligase, which in turn recognizes and binds to the protein to be degraded, catalyzing the transfer of activated ubiquitin from the E2 enzyme to the protein (ubiquitin is covalently bound to lysine residues of target proteins).^50^, ^51^ This protein, through the addition of several ubiquitin units, is poly‐ubiquitinated, and upon recognition by the 26S proteasome, it is degraded.^52^, ^53^ Therefore, the UPS is essential for maintaining cellular homeostasis, by eliminating denatured, damaged, mutated, and otherwise unneeded proteins.^54^, ^55^

**FIGURE 2:**
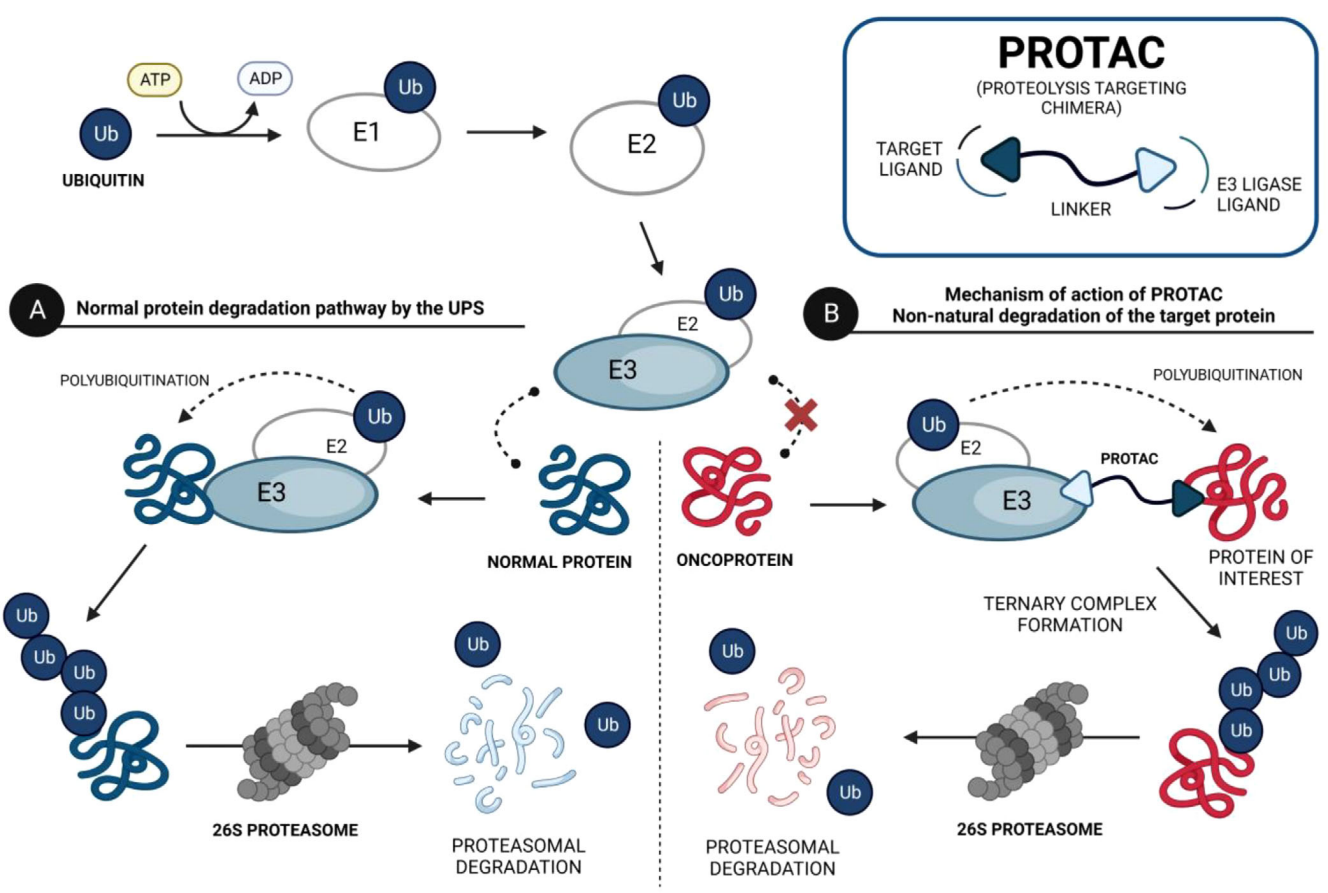
Ubiquitin–proteasome system (UPS) and the mechanism of action of PROteolysis‐Targeting Chimeras (PROTACs). (A) In the normal protein degradation pathway by the UPS, ubiquitin is activated by the E1–ubiquitin activating enzyme, in an ATP‐dependent process, and then transferred to the E2–ubiquitin conjugating enzyme. The E2–Ub complex then binds to the E3–ubiquitin ligase enzyme, which in turn binds to the protein to be degraded. E3 ligase catalyzes the transfer of ubiquitin units to the surface of the target protein, poly‐ubiquitinating it. Thus, it is subsequently recognized by the 26S proteasome and degraded, originating small peptide fragments. (B) In the non‐natural degradation of the target protein by a PROTAC, when inside the cell it simultaneously binds to the protein of interest (e.g., oncoproteins) and an E3 ligase, forming a stable ternary complex. This approximation between the E3 ligase and the protein, forced by PROTAC, results in the non‐natural poly‐ubiquitination of the target, and consequently in its degradation by the 26S proteasome.^46^, ^50^, ^58^

Structurally, PROTACs are molecules that can be divided into three pieces. At one end is a ligand capable of binding to the POI—the target ligand moiety (TLM). At the opposite end is a ligand capable of hijacking a specific E3 ligase—the E3 ligase moiety (ELM). These two pharmacophoric units are joined together by a linker.^56^, ^57^ Because of this chemical structure, PROTACs are referred to as heterobifunctional molecules or chimeric molecules capable of inducing non‐natural degradation of a target protein.^46^ Mechanistically, when PROTAC enters into the cell, it binds to the POI at the TLM‐containing end, and to the E3 ligase at the opposite ELM‐containing end, thus forming a stable ternary complex (POI–PROTAC–E3 LIGASE).^48^ By forming this ternary complex, PROTAC promotes a forced approximation between the target protein and the E3 ligase, resulting in the proximity‐induced polyubiquitination of the target, which is then recognized and degraded by the 26S proteasome (Figure 2).^46^, ^58^ With this revolutionary mechanism of action, PROTACs have gained great importance in the field of drug discovery, as they allow the transition from an inhibitor‐based paradigm to a degradation‐based paradigm, which has brought great advantages.^59^ One of the major advantages of PROTACs is that they allow the degradation of proteins that do not have an active site (e.g., transcription factors [TFs]), known as “undruggable proteins”, because they cannot be inhibited by conventional molecular inhibitors due to the absence of an active site.^46^ Since PROTAC only needs to bind to the surface of the POI and bring it close, temporarily, to the E3 ligase for degradation, it is able to interact with these types of proteins, opening the door to a variety of potential new therapeutic targets.^59^ Another great advantage of PROTACs is that they can present a catalytic mechanism of action (event‐driven mechanism) if they do not bind irreversibly to POI, allowing their use in substoichiometric concentrations, that is, one PROTAC molecule degrades several POI molecules.^48^ On the other hand, inhibitors present an occupancy‐based mechanism, which requires high concentrations to achieve a certain level of inhibition, and which is often responsible for the occurrence of adverse effects.^48^ Furthermore, several studies indicate that PROTACs, in addition to not causing compensatory expression of POI, not even intracellular accumulation, manage to promote the degradation of many of the mutant proteins conferring resistance to conventional inhibitors.^56^

**FIGURE 3:**
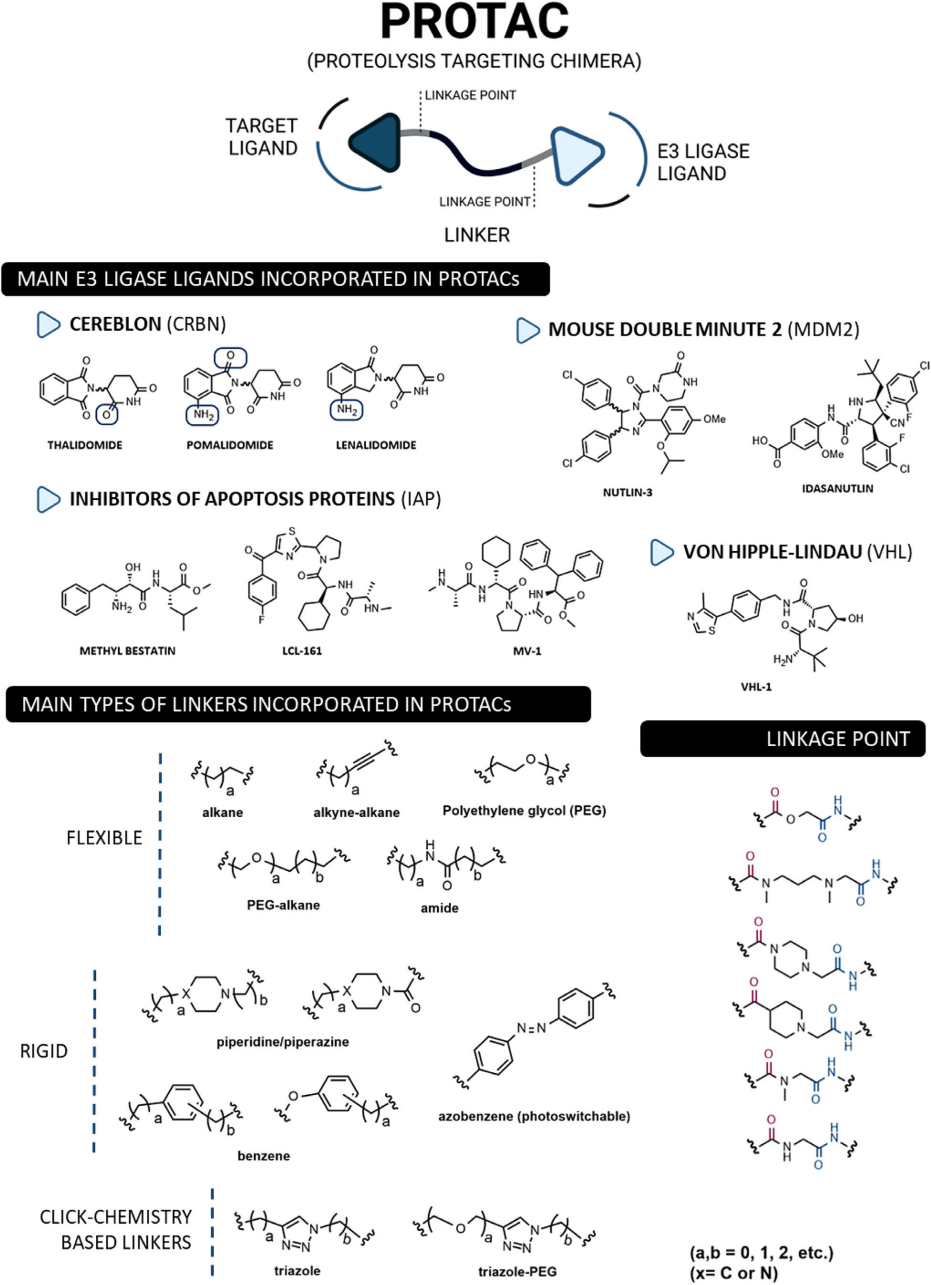
Structure and main building blocks of PROteolysis‐TArgeting chimeras (PROTACs). Structure of PROTACs, main E3 Ligase ligands, types of linkers incorporated in PROTACs and examples of the linkage points between the liker and the target ligand or E3 ligase ligand.

PROTACs present high versatility, as there is an enormous variety of targets, TLMs, and E3 ligase ligands, as well as linkers by varying their composition and length, that can be incorporated to form new compounds (Figure 3).^56^, ^57^ Over the years, countless human proteins have been targeted by PROTACs (more than 280 targets according to PROTAC‐DB) for the treatment of the most varied disorders, with emphasis on cancer, neurodegenerative, cardiovascular, immunological, or infectious diseases.^60^, ^61^ The linker is the part responsible for establishing the chemical connection between the TLM and the ELM. From flexible linkers (such as polyethylene glycol (PEG)‐based linkers or alkyl‐based linkers) to more rigid linkers, with cyclic structures, there is a huge variety of options.^46^, ^56^ However, this building block strongly influences the activity of PROTAC, since small changes in its composition, length, lipophilicity, flexibility, among other factors, affect not only the activity, but also the selectivity or pharmacokinetic properties of the degrader.^46^, ^48^ On the other hand, the number of E3 ligases recruited remains small compared with the large number of possibilities.^57^ The vast majority of PROTACs currently recruit one of the following four E3 ligases—Cereblon (CRBN), Von Hippel‐Lindau (VHL), mouse double minute 2 (MDM2), or cellular Inhibitor of Apoptosis Protein (IAP).^60^ Currently, more than 600 E3 ligases are known, indicating that there is still much to be explored.^46^

As mentioned previously, the activity of PROTAC is strongly influenced by the chosen combination of the TLM, ELM, as well as the linker, since all three of these pieces are decisive in ensuring the desired interaction between the POI and the E3 ligase, which culminates in the poly‐ubiquitination of the target.^60^ Thus, to identify a successful PROTAC, a set of generic studies are essential, such as:

- binding studies to evaluate the ability of the degrader to bind to POI and E3 ligase^60^;
- dose–response studies to determine the concentration conferring half‐maximal target degradation (DC_50_) and maximum degradation (*D*_max_)^61^;
- time‐course studies to evaluate POI degradation levels at different time points^61^;
- competition studies to evaluate the effect of coadministration of PROTAC with an inhibitor of the target, or the E3 ligase, or the proteasome on the activity of the degrader, and thus evaluate, for example, whether the degradation is proteasome or E3 ligase dependent^60^, ^61^;
- cell proteomics studies in order to evaluate the PROTAC's selectivity, that is, to evaluate whether PROTAC degrades only the desired target, or whether it interacts with other proteins present in the environment^60^;
- in PROTACs for cancer treatment, it is still essential to evaluate their anticancer activity in vitro, ex vivo, and in vivo.

Based on this information, for example, the ideal antileukemic PROTAC is one that has selective cytotoxic activity against leukemic cells, through the formation of a stable ternary complex (POI–PROTAC–E3 ligase), degrading completely, quickly, selectively, potently, and long‐lasting the target, with the lowest possible DC_50_ value, thus being effective and safe to be used in the clinic.

With more than 25 PROTACs in clinical trials, research continues to focus on obtaining new PROTACs with the previously mentioned characteristics. However, new challenges have also guided the progress of more recent PROTACs research. The first challenge is to expand the range of targets and E3 ligases that can be recruited, as well as moving from a trial‐and‐error model to a rational design of PROTACs, with the help of in silico tools and artificial intelligence.^57^, ^60^ The second challenge is to reduce the size of these compounds, since they are large and do not follow the Lipinski rule‐of‐five, which could compromise their future oral administration.^48^, ^60^ Furthermore, issues related to its solubility, stability, and cellular permeability still need to be clarified. Another important issue is to evaluate the pharmacokinetic properties of these compounds, as well as the behavior of the metabolites generated in vivo.^60^

Although there are many challenges ahead, PROTACs are already an undeniably powerful tool that will allow us to significantly reduce the “undruggable genome” and, as a new emerging therapeutic modality, will offer exciting potential for the future treatment of many different diseases.

## 3 PROTACS AGAINST LEUKEMIA‐ASSOCIATED TARGETS

The awakening of global interest in PROTACs is undeniable, whether in the pharmaceutical industry, research centers, or academia.^43^, ^62^ Currently, numerous PROTACs reported in the literature have demonstrated the ability of promoting selective degradation of specific targets involved in the development and progression of different types of leukemia. However, the overwhelming majority of antileukemic PROTACs are still in preclinical development. Nonetheless, there are some promising degraders capable of effectively impairing the target, indicating potential for future treatment of one or more types of leukemia. The proteins Breakpoint Cluster Region Abelson (BCR‐ABL), BCL‐XL, Bruton's tyrosine kinase (BTK), Bromodomain‐containing protein 4 (BRD4), cyclin‐dependent kinases (CDK6), FMS‐like tyrosine kinase 3 (FLT‐3), among others, have been identified as the main targets for degradation by antileukemia PROTACs.^6^, ^63^, ^64^

**FIGURE 4:**
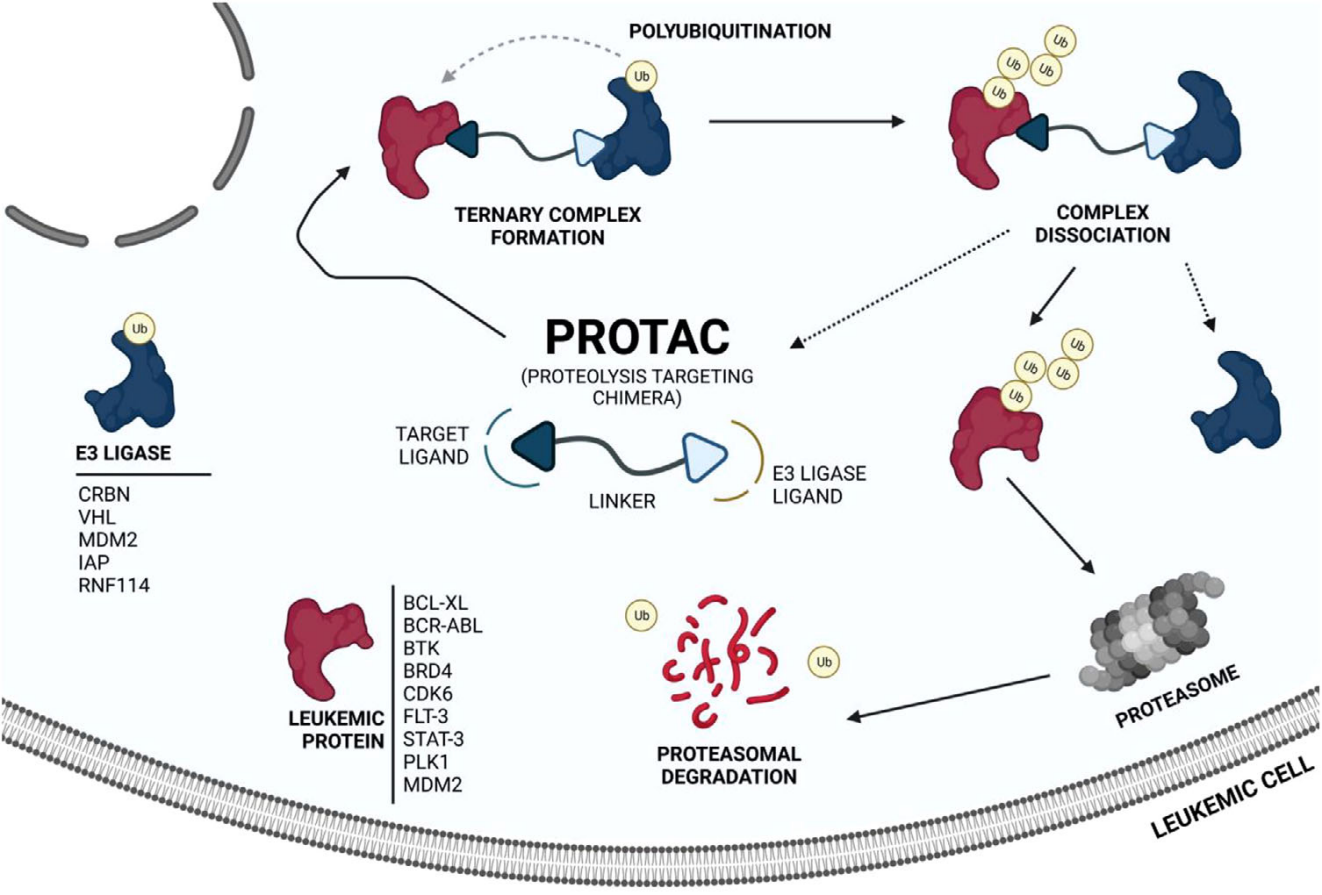
Mechanism of PROteolysis‐TArgeting Chimeras (PROTACs) against leukemia‐associated targets and main E3 ligases recruited. When a PROTAC capable of binding to a leukemia‐associated protein enters a leukemic cell, it forms a stable ternary complex between the target protein and the recruited E3 ligase, leading to polyubiquitination and subsequent degradation of the leukemia‐associated protein by the 26S proteasome. When the antileukemic PROTACs do not irreversibly bound to the target, the dissociation of the complex occurs, and the PROTACs can bind to a new target protein and promote its polyubiquitination, having a catalytic activity (event‐driven mechanism). The main E3 ligases recruited by antileukemic PROTACs are cereblon (CRBN), Von Hippel‐Lindau (VHL), mouse double minute 2 (MDM2), and cellular inhibitor of apoptosis protein (IAP).^63^, ^64^

Several studies demonstrated that some PROTACs have achieved potent, rapid, selective, and time‐sustained degradation of the intended targets in vitro and in vivo. Below is a critical analysis and discussion on PROTACs targeting key leukemia‐associated targets (Figure 4) as reported in the scientific literature. These PROTACs are currently under study for the treatment of one or more types of leukemia and are presented here by therapeutic target and chronological order.

### 3.1 BCR‐ABL

BCR‐ABL is a protein that results from the chromosomal translocation of the ABL gene from chromosome 9 to the BCR gene on chromosome 22, t(9;22), originating the so‐called Philadelphia chromosome (Ph), present in most cases of CML (>95%), and in some cases of ALL (ALL‐Ph^+^).^65^, ^66^, ^67^ As a result of this chromosomal translocation in HSCs, the oncogenic fusion protein BCR‐ABL is expressed. This protein possesses constitutive tyrosine kinase action, which aberrantly activates a series of signaling pathways that promote cell proliferation, such as the Janus kinase/signal transducer and activator of transcription 5 (STAT5), phosphatidylinositol‐3‐kinase/Akt, mitogen‐activated protein kinase, and CrkL signaling pathways.^68^, ^69^ This abnormal activation results in intracellular deregulations and reprogramming (e.g., changes in cell adhesion, migration, and survival) that lead to an uncontrolled overproduction and expansion of leukemic cells, culminating in the progressive development of malignant hematologic diseases.^68^, ^70^

#### 3.1.1 Inhibitors of BCR‐ABL

Over the past few, several researchers have focused on developing compounds capable of binding to the ATP binding site of the kinase domain of BCR‐ABL, and thus inhibiting this oncoprotein. Currently, there are several tyrosine kinase inhibitors (TKIs) approved for treating CML, classified according to their potency and activity against the various mutant forms of BCR‐ABL. The first was imatinib (IMA), followed by the second generation—dasatinib (DAS), nilotinib (NIL), and bosutinib (BOS)—and the third generation, ponatinib (PON).^71^ More recently, allosteric inhibitors of BCR‐ABL, such as asciminib (ASC) and GNF‐5, have been designed. These inhibitors bind to the myristoyl binding pocket in the kinase domain, inactivating BCR‐ABL activity by preventing its bioactive conformation.^72^, ^73^ However, treatment with TKIs requires lifelong administration due to the persistent leukemic stem cells (LSCs) resulting from BCR‐ABL's scaffold protein functions, which activate kinase‐independent pathways.^74^ In addition to the adverse effects resulting from the administration of these TKIs, there is also the possibility of resistance occurring that may preclude the use of these types of inhibitors, particularly the T315I mutation.^71^

#### 3.1.2 PROTACs against BCR‐ABL

While recent, the study of new PROTACs targeting BCR‐ABL has gained significant attention, not only due to the number of scientific articles published, but also due to increasing number of patent applications submitted in recent years. This is a clear indication of the promising potential of these degraders for the treatment of CML.^75^

In a communication published at the end of 2015, Crews’ group presented, for the first time, a library of PROTACs targeting the BCR‐ABL protein, recruiting either CRBN or VHL E3 ligases.^76^ To construct these PROTACs, three different TKIs—IMA, BOS, and DAS—were conjugated to ELM, using four different types of linkers, varying in their composition and length.^76^ After testing the PROTACs obtained in K562 CML cells, it was found that all IMA‐based PROTACs, as well as all VHL ligase‐recruiting PROTACs, were unable to degrade the BCR‐ABL protein.^76^ By exchanging the VHL ligand for a CRBN ligand (pomalidomide), both DAS‐CRBN and BOS‐CRBN PROTACs degraded BCR‐ABL, with a degradation of more than 60% at 1 μM and more than 80% at 2.5 μM, respectively.^76^ In cell viability studies, the most potent PROTAC (DAS‐6‐2‐2‐6‐CRBN (1) (Table 2)), capable of degrading BCR‐ABL up to 25 nM, demonstrated nanomolar potency in BCR‐ABL driven K562 cells.^76^ In contrast, in cell viability studies in non‐BCR‐ABL driven cells (e.g., HEK293T and SK‐BR‐3 cells), PROTAC DAS‐6‐2‐2‐6‐CRBN was found to have a 103‐fold lower activity compared with that observed in BCR‐ABL driven cells (K562 cells). This indicates that the current degrader demonstrated significantly more selective activity against the BCR‐ABL driven cell line K562. This selective action is advantageous, as it may reduce the possibility of adverse effects resulting from PROTAC activity in other cells (off‐target effects).^76^

**TABLE 2:** Chemical structure of PROTACs targeting BCR‐ABL protein.

No.	Chemical structure	PROTAC Information	References
1	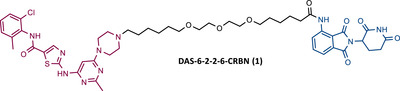	TLM: Dasatinib ELM: Cereblon ligand (pomalidomide) DC_50_: NA	76
2	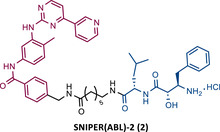	TLM: Imatinib ELM: IAP ligand DC_50_: NA	77
3	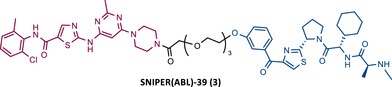	TLM: Dasatinib ELM: IAP ligand DC_50_: NA	78
4	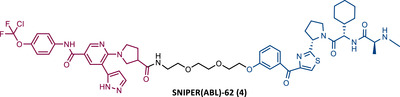	TLM: Asciminib (ABL‐001) ELM: IAP ligand DC_50_: NA	79
5	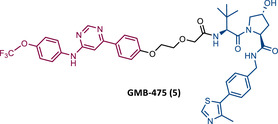	TLM: GNF‐5 ELM: VHL ligand DC_50_ = 340 nM (K562 cells) IC_50_ = 1.11 μM (Ba/F3 cells)	80
6	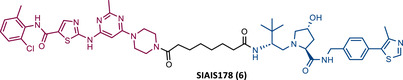	TLM: Dasatinib ELM: VHL ligand DC_50 _= 8.5 nM (K562 cells) IC_50_ = 24 nM (K562 cells)	81
7	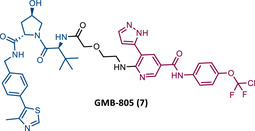	TLM: Asciminib (ABL‐001) ELM: VHL ligand DC_50 _= 30 nM (K562 cells) IC_50_ = 169 nM (K562 cells)	82
8	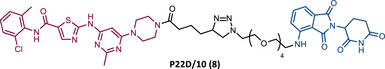	TLM: Dasatinib ELM: Cereblon ligand (pomalidomide) DC_50 _= 10 nM (K562 cells)	83
9	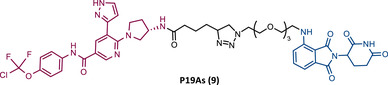	TLM: Asciminib (ABL‐001) ELM: Cereblon ligand (pomalidomide) DC_50 _= 200 nM (K562 cells)	83
10	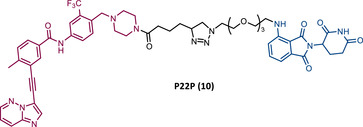	TLM: Ponatinib ELM: Cereblon ligand (pomalidomide) DC_50 _= 20 nM (K562 cells)	83
11	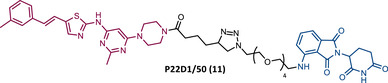	TLM: Dasatinib derivative ELM: Cereblon ligand (pomalidomide) DC_50_: NA	83
12	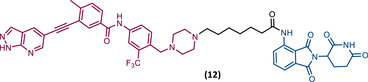	TLM: GZD824 ELM: Cereblon ligand (pomalidomide) DC_50 _= 109 nM (Ba/F3^T315I^ cells) IC_50 _= 27 nM (Ba/F3^T315I^ cells)	84
13	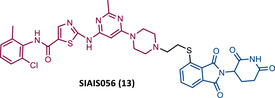	TLM: Dasatinib ELM: Cereblon ligand (pomalidomide) DC_50 _= 0.18 nM (K562 cells) IC_50_ = 0.49 nM (K562 cells)	85
14	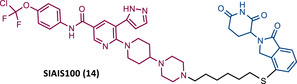	TLM: Asciminib (ABL‐001) ELM: Cereblon ligand (lenalidomide derivative) DC_50 _= 2.7 nM (K562 cells) IC_50_ = 12 nM (K562 cells)	86

*Note*: 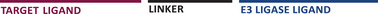

Abbreviations: IAP, cellular inhibitor of apoptosis protein; ELM, E3 ligase moiety; NA, not applicable; TLM, target ligand moiety; VHL, Von Hippel‐Lindau.

In the following period, the first IAP‐based PROTACs, also known as specific and nongenetic IAP‐dependent protein erasers (SNIPERs), targeting BCR‐ABL were designed. Naito et al.^77^, ^78^, ^79^ studied these degraders and developed SNIPERs capable of degrading BCR‐ABL with micro to nanomolar potency. Examples include SNIPER(ABL)−2 (2), SNIPER(ABL)−39 (3), or SNIPER(ABL)−62 (4), which have been shown to be effective in CML cells.^77^, ^78^, ^79^ However, these studies lacked in‐depth analysis of SAR studies, or the activity of PROTACs in cell lines with mutations in the BCR‐ABL gene.

In 2019, Crews et al. introduced a new series of BCR‐ABL based PROTACs.^80^ These PROTACs present as TLM an allosteric inhibitor of BCR‐ABL (GNF‐5) and as ELM a VHL ligand.^80^ The lead compound, GMB‐475 (5), induced a significant and rapid degradation of BCR‐ABL in a time‐ and concentration‐dependent manner, inhibiting cellular proliferation of human CML cells with micromolar potency (50% inhibitory concentration (IC_50_) value of 1 μM), but not as potent as IMA (IC_50 _= 0.17 μM).^80^

The introduction of T315I or G250E mutations into Ba/F3 cells led to a reduction in IMA activity, however, these cells remained susceptible to the effects of GMB‐475 (IC_50_ values of 1.98 and 0.37 μM, respectively).^80^ Cotreatment with GMB‐475 and IMA demonstrated a threefold reduction in the IC_50_ of the inhibitor, proving that the concerted use of degrader with orthosteric TKIs could reduce adverse events associated with the use of the latter.^80^

Furthermore, in in vitro studies with primary CML‐CD34^+^ patient cells, PROTAC GMB‐475 reduced cell viability and induced apoptosis.^80^ In healthy donor CD34^+^ cells, the degrader did not demonstrate any effect.^80^ However, in primary patient LSCs, it induced the degradation of BCR‐ABL.^80^ This study thus demonstrated that VHL‐based PROTACs targeting BCR‐ABL are active when targeted to the allosteric site, and active against BCR‐ABL mutant forms.

That same year, Zhao et al.^81^ presented for the first time a potent VHL‐based PROTAC targeting BCR‐ABL, SIAIS178 (6), which incorporated DAS as TLM. This finding contradicted the previously held notion that an orthosteric VHL‐based PROTACs was not suitable for the degradation of BCR‐ABL.^81^ Although Crews had previously introduced some DAS‐VHL PROTACs, none of them were able to degrade the target.^81^

However, Zhao's study began with the design of several PROTACs, which link DAS to the VHL‐1 ligand, through different types of linkers (PEG‐based linkers and carbon chain linkers), and subsequent evaluation of their degradative activity in K562 cells.^81^ The results showed that all PROTACs with PEG‐based linkers do not degrade POI up to 10 μM.^81^ By changing the linker to carbon chains, PROTACs effectively degraded BCR‐ABL (5–10 carbon atoms), especially SIAIS178, with DC_50_ and IC_50_ values of 8.5 and 24 nM, respectively.^81^

This demonstrates that, in this case, intraoxygenated linkers have a negative impact on the activity of PROTACs that bind to the ATP‐binding site. This is unrelated to VHL's inability to degrade the target but rather to the impact of the linker on the formation of the ternary complex.

Studies with PROTAC SIAIS178 demonstrated that it had a significant antiproliferative effect on BCR‐ABL driven cells, although lower than that obtained with DAS alone.^81^ It showed good selectivity by interacting with fewer targets than DAS, and it did not present cytotoxic effects on non‐BCR‐ABL leukemia cells.^81^ In vivo, PROTAC SIAIS178 degraded BCR‐ABL and consequently presented a potent antileukemic effect in the murine xenograft model of K562 cells.^81^

When tested on several clinically relevant mutant isoforms that confer resistance to IMA or DAS, PROTAC SIAIS178 showed the ability to degrade some of the mutant forms of BCR‐ABL. However, mutations at position T315 that affect the direct binding of DAS to BCR‐ABL may also compromise the degrader's activity.^81^ Another advantage highlighted in studies with PROTAC SIAIS178 is that its antileukemic action persists longer after its removal compared with its warhead relative.^81^

In 2020, Crews’ group reported its second allosteric VHL‐based PROTAC targeting BCR‐ABL, designated GMB‐805 (7).^82^ Although very similar to its predecessor GMB‐475, this one features the allosteric inhibitor Abl‐001 as its TLM.^82^ This change resulted in a PROTAC with a capacity to promote target degradation 10 times greater, with a DC_50_ value of 30 nM, compared with the DC_50_ value of 340 nM of GMB‐475.^82^ In in vitro studies, this new PROTAC reduced cell proliferation in CML cells (IC_50 _= 169 nM) and demonstrated activity in vivo.^82^ However, more studies to evaluate its activity are still awaited.

In the same year, Rao and colleagues presented an extensive library of new CRBN‐based PROTACs synthesized through click chemistry. These PROTACs result from connecting a TKIs (IMA, DAS, ASC, or PON) to pomalidomide, with a PEG‐based linker.^83^ From Western blot analysis on K562 cells, it was confirmed that IMA‐based PROTACs did not induce degradation of BCR‐ABL.^83^ The most powerful DAS‐based PROTAC P22D/10 (8) exhibited similar degradative potency as previously reported DAS‐PROTACs (DC_50 _= 10 nM). However, it was susceptible to the T315I mutation.^83^ The ASC‐based PROTAC P19As (9) had slight degradation potency (DC_50 _= 200 nM), and PON‐based PROTAC P19P (10) had similar potency to DAS‐based PROTAC (DC_50 _= 20 nM).^83^

However, despite being more selective and safer, all degraders had less activity than that shown by the corresponding parent warhead.^83^ Only PROTACs P19As and P19P were capable of degrading the T315I mutation, with DC_50_ values higher than those observed in wild‐type (WT) CML cells, due to the reduction in binding affinity.^83^ Furthermore, this group studied the impact that replacing the amide bond in DAS with a hydrophobic group has on the performance of PROTAC.^83^ By incorporating this new derivative of DAS as TLM, PROTAC P22D1/50 (11) was constructed, which degraded BCR‐BL T315I at concentrations below 1 μM, and with better antiproliferative activity than DAS.^83^ With this study, it is also possible to conclude that for PROTACs recruiting CRBN, the ATP‐binding pocket of BCR‐ABL may be the most effective binding site.

The BCR‐ABL^T315^ gatekeeper mutation has been a major concern in the treatment of CML.^72^ In response to this unmet clinical challenge, Lu's group presented the PROTAC 12 with an IC_50_ value of 27 nM against Ba/F3^T315I^ cells.^84^ PROTAC 12, has an inhibitor GZD824 (phase II candidate) as the TLM and pomalidomide as the ELM, linked through a six‐member carbon chain linker, capable of degrading BCR‐ABL^T315I^ with a DC_50_ value of 109 nM.^84^ Furthermore, it showed activity in vivo by reducing tumor growth in Ba/F3^T315I^ xenograft tumor models.^84^

In 2021, Jiang's group, which previously reported the VHL‐based PROTAC SIAIS178, introduced a new DAS‐based PROTAC recruiting CRBN designated SIAIS056 (13).^81^, ^85^ From their extensive SAR studies with different CRBN‐based PROTACs with DAS, it can be concluded that, first, the length of the linker, whether PEG‐based or alkyl‐based, does not appear to be a relevant factor in the activity of the PROTAC, although it does affect the pharmacokinetic properties in vivo^85^; second, the acyl substitution at the ends of the linker reduces the potency of the degrader, which is increased in the presence of an alkylated conjugation between the linker and DAS and the CRBN ligands^85^; third, sulfur‐substituted lenalidomide or pomalidomide at position C‐4 are more potent in degrading BCR‐ABL and with better antiproliferative activity.^85^

The most powerful degrader, SIAIS056, demonstrated that it can selectively degrade WT BCR‐ABL and some BCR‐ABL mutations, but not T315I, with nanomolar potency, reducing the activation of the respective signaling pathway.^85^ It should also be noted that in vitro, PROTAC 13 was more potent than the VHL‐based PROTAC SIAIS178.^85^ In vivo, PROTAC 13 demonstrated antileukemic activity.^85^

In 2022, this same group presented SIAIS100 (14), a new CRBN‐based PROTAC targeting the myristoyl‐binding pocket of BCR‐ABL, as it incorporates the allosteric inhibitor ASC.^86^ From the SAR studies, it was again confirmed that the use of PEG‐based linkers reduces the activity of this series of PROTACs, being favored by carbon linkers.^86^ The incorporation of CRBN ligands substituted at the C‐4 position benefits PROTAC activity.^86^ Additionally, replacing piperazine with a bicyclic piperidinyl‐piperazine group made it possible to obtain the potent PROTAC 14 (DC_50 _= 2.7 nM and *D*_max _= 91.2%), with good antiproliferative activity (IC_50_ = 12 nM) in K562 cells. However, this activity was lower than that obtained with ASC, possibly due to lower cell permeability and degrader binding affinity.^86^ It is important to note that PROTAC 14 was selective and caused a significant reduction in the levels of the T315I and E255V mutant forms, as well as some ASC‐resistance mutations.^86^

One of the major challenges in the development of PROTACs has been the possibility of temporally and spatially controlling their activity, in order to obtain maximum efficacy and safety.^87^ Currently, there are already PROTACs that incorporate trigger or switch modules, enabling control over their activity.^87^ For example, the incorporation of a photoswitch module, such as an azobenzene (Azo) group, allows rapid and reversible control of some compounds.^87^ Indeed, in 2020, Jin et al.^88^ published an article in which they presented a pioneer controllable PROTAC targeting BCR‐ABL called Azo‐PROTAC‐4C (15). To construct this PROTAC, DAS was linked via an alkyl‐linker with four carbon atoms to an azo unit, in turn, attached at the 3‐position of the phenyl group in lenalidomide (CRBN ligand).^88^ Azo‐PROTAC‐4C demonstrated that it can induce the CRBN‐dependent degradation of BCR‐ABL, thereby selectively inhibiting the cellular proliferation of CML cells with nanomolar potency (IC_50 _= 68 nM).^88^

**FIGURE 5:**
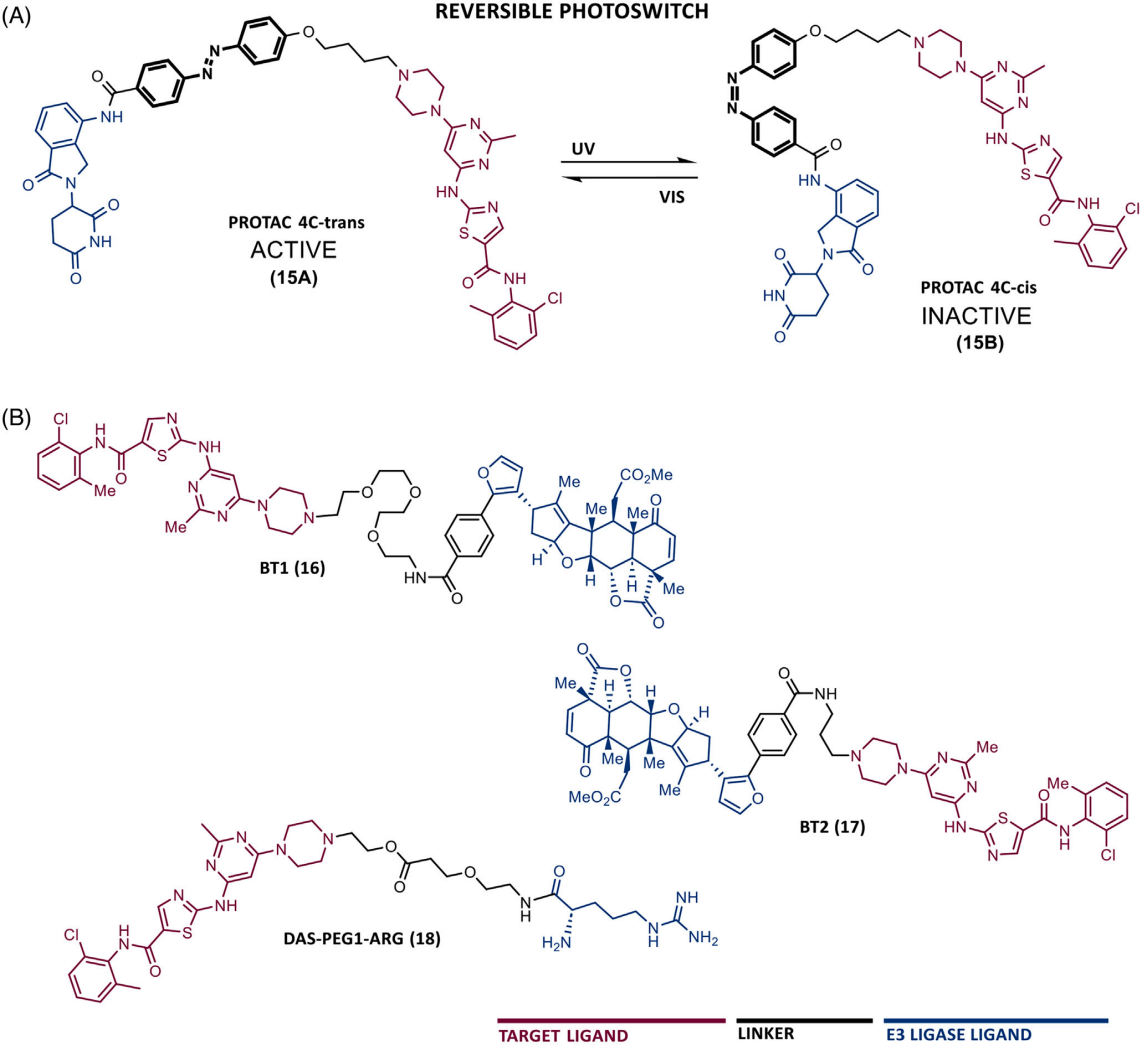
Chemical structure of PROTACs targeting BCR‐ABL. (A) Reversible photoswitch mechanism of BCR‐ABL PROTAC 15. (B) Chemical structure of PROTACs targeting BCR‐ABL by recruiting less common recruited E3 ligases.

However, the cis‐ and trans‐configurations of this degrader do not have the same ability to induce degradation of the fusion protein.^88^ In reality, only 4C‐trans‐configuration degraded BCR‐ABL, with 90% degradation after 32 h of treatment.^88^ Knowing that it is possible to change the configuration of azo‐PROTAC with UV‐C light (changing from a trans (15A) to a cis‐configuration (15B)) (Figure 5), it was observed that in live CML cells treated with PROTAC 4C‐trans, that irradiation with UV‐C radiation caused an increase in BCR‐ABL protein levels.^88^ Therefore, these studies demonstrated the possibility of reversibly controlling the degradation of BCR‐ABL induced by the photoswitchable azo‐PROTAC by changing its configuration by UV‐C light.

In addition to PROTACs recruiting VHL or CRBN E3 ligases, there are also some examples of PROTACs recruiting MDM2 or RNF114 ligases.^89^, ^90^ Recruiting other E3 ligases in the design of new PROTACs is advantageous since it hinders the potential occurrence of resistance to degraders on the part of the E3 ligase.

In 2020, two PROTACs were reported resulting from linking DAS with an RNF114 E3 ligase ligand, nimbolide, through a PEG linker (BT1 (16)) or a short alkyl linker (BT2 (17)).^90^ Of note is BT1, the first and most potent RNF114‐based PROTAC targeting BCR‐ABL to date, which has been shown to degrade the target and to have additional antileukemic effects by increasing the levels of the tumor suppressor p21 in CML cells.^90^

More recently, PROTAC ^PMI^BCR/ABL‐R6 was reported, as the first peptide PROTAC and also the first to recruit the MDM2 E3 ligase targeting BCR‐ABL.^89^ This PROTAC presents as ELM, an MDM2 inhibitor called PMI, and as TLM, a peptide moiety capable of binding to the oligomerization domain of BCR‐ABL.^89^ A poly‐arginine tail was added to this structure to increase cell permeability.^89^ Theoretically, this PROTAC has enormous potential. First, in general, peptide drugs have better affinity and specificity for the target.^89^ Second, this degrader is capable of being active against the common drug resistance mutations of BCR‐ABL since it does not bind to the ATP‐biding site.^89^ Third, in addition to inducing the degradation of BCR‐ABL, this degrader, by recruiting MDM2, prevents the binding of this E3 ligase to the tumor suppressor p53, thus increasing the levels of the suppressor.^89^ All this indicates that this peptide PROTAC has a dual mechanism of action. In vitro and in vivo studies demonstrated that this degrader is selective and has activity, with a dual effect, even in situations of resistance to IMA, surpassing the results obtained with IMA or nutlin (MDM2 inhibitor) alone.^89^

In 2023, Rao et al.^91^ expanded the toolbox of the E3 ligases and ligands effective for PROTACs, by designing a new typology of PROTACs, called single amino acid‐based PROTACs, targeting BCR‐ABL. These degraders present as ELM a single destabilizing amino acid that is recognized by a UBR E3 ligase, promoting target degradation via the N‐end rule pathway.^91^ Therefore, PROTACs were synthesized with DAS as the TLM, linked by a PEG‐based linker, to a destabilizing amino acid (arginine, leucine, lysine, or phenylalanine).^91^ When tested on CML cells, these PROTACs degraded BCR‐ABL and exhibited an antiproliferative effect with nanomolar potency.^91^ It is worth noting that the results obtained with this type of PROTAC presented IC_50_ and DC_50_ values lower than most previous PROTACs, demonstrating that the use of N‐end rule‐based PROTACs allows to obtain degraders with lower molecular weight, potent and higher duration of action.^91^ Among all, PROTAC arginine‐PEG1‐DAS (18) (DC_50 _= 0.85 nM, *D*_max _= 98.8%) stands out, demonstrating good antileukemic activity both in vitro and in K562 xenograft mouse model in vivo.^91^

### 3.2 BCL‐2 family

Consisting of a series of pro‐ and antiapoptotic proteins, the B‐cell lymphoma 2 (BCL‐2) family proteins, play a fundamental role in controlling the cell life cycle.^92^, ^93^ Most notably, the antiapoptotic proteins BCL‐2, B‐cell lymphoma extra‐large (BCL‐XL), and myeloid leukemia‐1 (MCL‐1) are frequently overexpressed in various types of cancer. Their presence leads to evasion of the apoptosis process, thereby promoting the initiation and tumor progression, and the development of resistance.^94^, ^95^ Through their binding to the α‐helical BCL‐2 homology‐3 (BH3) domain of proapoptotic proteins Bax and Bak, they prevent the activation of the mitochondrial apoptotic pathway.^96^, ^97^

#### 3.2.1 Inhibitors of BCL‐2 family

The study of “BH3 mimetic” SMIs has led to the development of inhibitors, such as venetoclax (an United States Food and Drug Administration [US FDA]‐approved BCL‐2 specific‐inhibitor for CLL and AML), navitoclax (BCL‐2/BCL‐XL dual inhibitors), or BCL‐XL specific inhibitors.^98^, ^99^ The BCL‐XL protein is a prominent protein in several types of leukemia, where it is overexpressed, and known to promote drug resistance.^100^ However, given the dependence of platelet survival on BCL‐XL, the use of SMIs targeting this protein is often associated with the occurrence of on‐target and dose‐limiting thrombocytopenia toxicity.^99^, ^100^, ^101^

#### 3.2.2 PROTACs against BCL‐2 family

In 2019, two distinct groups reported different and selective BCL‐XL PROTACs (Table 3).^99^, ^100^

**TABLE 3:** Chemical structure of PROTACs targeting BCL‐XL protein.

No.	Chemical structure	PROTAC information	References
19	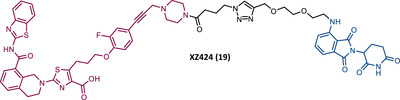	TLM: BCL‐XL ligand ELM: Cereblon ligand (pomalidomide) DC_50 _= 50 nM (MOLT‐4 cells) IC_50 _= 51 nM (MOLT‐4 cells)	100
20	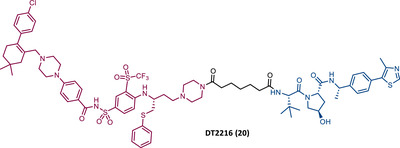	TLM: Navitoclax ELM: VHL ligand DC_50_ = 63 nM (MOLT‐4 cells)	99
21	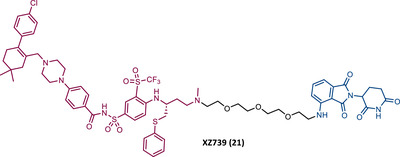	TLM: Navitoclax ELM: Cereblon ligand (pomalidomide) DC_50 _= 2.5 nM (MOLT‐4 cells) IC_50 _= 10.1 nM (MOLT‐4 cells)	102
22 23 24	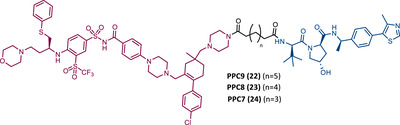	TLM: Navitoclax ELM: VHL ligand DC_50_ < 30 nM (293T cells)	103
23A	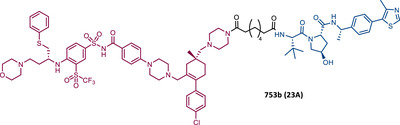	TLM: Navitoclax ELM: VHL ligand DC_50 _= 6 nM (293T cells)	103
25 26 27	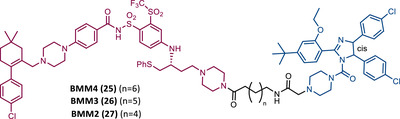	TLM: Asciminib (ABL‐001) ELM: MDM2 ligand (Nutlin‐3) DC_50_: NA	105

*Note*: 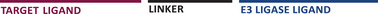

Abbreviations: ELM, E3 ligase moiety; MDM2, mouse double minute 2; NA, not applicable; TLM, target ligand moiety; VHL, Von Hippel‐Lindau.

Zheng's group reported the compound XZ424 (19), the first pomalidomide‐based PROTAC targeting BCL‐XL, synthesized via click chemistry. When tested in BCL‐XL dependent ALL cells (MOLT‐4 cells) PROTAC XZ424 demonstrated to degrade the target in a time‐ and dose‐dependent manner, with a DC_50_ value of 50 nM. This effect was reversible and long‐lasting, resulting in cytotoxicity induced through caspase‐dependent apoptosis.^100^ In contrast, BCL‐XL levels in human platelets did not change significantly when exposed to the degrader.^100^ Cellular cytotoxicity studies demonstrated that PROTAC XZ424 is potently cytotoxic against BCL‐XL dependent ALL cells (MOLT‐4 cells), with an IC_50_ value of 51 nM.^100^ Furthermore, this cytotoxicity activity is about 22 times more selective for MOLT‐4 cells than for platelets.^100^ In contrast, specific BCL‐XL inhibitors are cytotoxic to both MOLT‐4 cells and platelets.^100^ Although the authors do not provide a proven explanation for this, the improved selectivity of XZ424 is probably due to the different degradation efficiency of BCL‐XL in MOLT‐4 compared with platelets.^100^ Therefore, this study demonstrated that the PROTAC approach can be a new way to achieve tissue selectivity, and thus reduce the occurrence of adverse effects, in this specific case, thrombocytopenia.

On the other hand, Zhou's group reported the compound DT2216 (20), the first VHL‐based PROTAC targeting BCL‐XL, and which incorporates the navitoclax inhibitor as TLM, linked by an alkyl‐based linker to a VHL ligand.^99^ In addition to this compound being cytotoxic against BCL‐XL dependent leukemia cell line (MOLT‐4 cells), with a DC_50_ value of 63 nM and a *D*_max_ value of ∼91%, it was shown to be less toxic to platelets than the respective TLM alone, given the low expression of VHL on platelets.^99^ However, it is necessary to consider that even though it does not degrade BCL‐XL in platelets, it has the ability to inhibit this protein, and therefore, is not free from causing thrombocytopenia. Selectivity studies demonstrated that PROTAC DT2216 only degrades BCL‐XL, not changing levels of BCL‐2 or MCL‐1 in the tested cells, as did the previous PROTAC XZ424.^99^ In vivo studies demonstrated that PROTAC DT2216 is less toxic to platelets than navitoclax, and with better results in suppressing the growth of MOLT‐4 T‐ALL xenografts in mice.^99^ Therefore, it is not only safer, but also more potent. Drug synergism studies demonstrated that the combination of PROTAC DT2216 with the inhibitor venetoclax makes it possible to efficiently treat BCL‐XL and BCL‐2 dependent tumors, without causing significant platelet toxicity.^99^ Furthermore, the degradation of BCL‐XL induced by the degrader may reduce chemotherapy resistance of malignant cells in vivo.^99^

In 2020, Zheng's group reported a diverse library of new degraders, consisting of four series of PROTACs that present the navitoclax inhibitor as TLM, in which the morpholine ring was replaced by a piperazine ring, linked by different types of linkers to VHL or CRBN E3 ligases.^102^ All of these PROTACs were tested on MOLT‐4 cells, to assess their cytotoxicity.^102^ From the analysis of the series of PROTACs that recruit VHL, whether those with linkers containing an amide linkage and an alkane chain, or those with intraoxygenated linkers, or those incorporating different linkers tethered through an N‐methylamino group of TLM to a VHL ligand, none of them managed to surpass the results obtained by PROTAC DT2216 (IC_50_ value of 77.1 nM, after 48 h treatment).^102^ By replacing the VHL ligands of compounds from the previous series with pomalidomide, a variety of new CRBN‐based PROTACs were designed.^102^ It should be noted that those with C–N linkage were generally more potent than their amide‐linkage counterparts, but generally, they all showed a positive correlation between the ability to deplete BCL‐XL and reduced MOLT‐4 cell viability.^102^ The lead compound, PROTAC XZ739 (21) (IC_50 _= 10.1 nM), with an 11‐atom PEG‐based linker, was approximately 22 times more potent than the respective TLM alone against MOLT‐4 cells, presenting a DC_50_ value of 2.5 nM, and a long‐lasting, reversible and dose‐dependent effect.^102^ PROTAC XZ739 showed 100 times greater selectivity for MOLT‐4 cells over platelets, while the navitoclax inhibitor presented cytotoxicity against both cell types.^102^ Western blot studies suggested that the apoptotic cell‐death mechanism of PROTAC XZ739 results from increased poly(ADP‐ribose) polymerase (PARP) and caspase‐3 cleavage in leukemic cells.^102^ It is worth noting that when MOLT‐4 cells were tested with 10 nM of PROTAC XZ739 there was no decrease in lymphoid TFs Ikaros (IKZF1) and Aiolos (IKZF3) protein levels, a common adverse effect of CRBN‐based PROTACs, promoted by immunomodulatory drugs (IMiDs), such as pomalidomide.^102^ However, treatment with 100 nM of PROTAC XZ739 demonstrated an induction of the IKZF1/3‐degradation.

In 2021, Zhou's group (the same group that in 2019 had reported the potent VHL‐based BCL‐XL PROTAC DT2216) demonstrated through computational modulation studies that of the four lysines present on the surface of BCL‐XL, only a single residue of lysine (K87) is accessible to the active site of the E2 enzyme in DT2216‐induced BCL‐XL degradation.^99^, ^103^ Furthermore, this study demonstrated that both the location and orientation of lysines are decisive for their accessibility to ubiquitin transfer.^103^

Associating these studies with the fact that PROTAC DT2216 has a limited effect on other types of leukemia than BCL‐XL‐dependent T‐ALL, Zhou's group has invested in developing a series of new VHL‐based PROTACs, which use the same TLM as the DT2216 (navitoclax).^103^ However, with a different linker attachment site—through one of the two methyl groups of the cyclohexene ring—in order to evaluate the impact on the geometry of the interaction between BCL‐XL and VHL, and consequently on the exposure of POI's lysines.^103^ In fact, powerful BCL‐XL/2 dual degraders (PPC7 to 9 (22‐24)) were obtained, with greater degradation ability of BCL‐XL than PROTAC DT2216 (^BCL‐XL^DC_50 _= 30 nM).^103^ Highlighting, the new PROTAC PPC8 (23), more specifically, its R‐epimer, designated 753b (23A), which exhibited the highest potency among all (^BCL‐2^DC_50 _= 48 nM and ^BCL‐XL^DC_50 _= 6 nM).^103^ Although PROTAC PPC8 formed the strongest ternary complex with both BCL‐XL and BCL‐2 proteins, and was consequently the most potent, its effect did not solely depend on the formation of this complex.^103^ In fact, among other factors, the exposure and orientation of lysines on the target surface are also critical factors.^103^ Of note, 753b‐induced BCL‐XL degradation is mediated by two lysines (K87 and K20), unlike DT2216 (only targets K87).^103^ Furthermore, 753b‐induced BCL‐2 degradation is dependent on lysine K17.^103^ Thus, demonstrating that changing the linker attachment site on the warhead, impacts the band region of surface lysines, and consequently affects both the selectivity and potency of the PROTAC. When compared with navitoclax alone, PROTAC 753b demonstrated, like the DT2216 degrader, to be less toxic to platelets (possibly due to a lower expression of VHL in these cells).^103^

Very recently, Konopleva's group published an article studying PROTAC 753b in more depth.^104^ In this article, in addition to proving that the 753b degrader has a potent antileukemia effect in primary patient‐derived AML blasts and in vivo activity in an AML‐derived patient‐derived xenograft model, it was found that it does not degrade BCL‐2 as readily as it degrades BCL‐xl.^104^ This discrepancy may be related not only to the lysine residues, but also to factors such as the protonation state, flexibility, or other amino acids in the vicinity of the ubiquitination zone.^104^ In this study, the senolytic properties of PROTAC 753b were also evaluated and indicated that chemotherapy‐induced senescent cells express higher levels of BCL‐XL.^104^ In this sense, the present PROTAC demonstrated to eliminate these BCL‐XL‐expressing senescent leukemia cells, thus increasing the effectiveness of chemotherapy.^104^ Furthermore, the activity of this degrader resulted in an increase in the expression of the MCL‐1 protein, which when used in conjunction with an MCL‐1 inhibitor induced synergistic leukemia cell death.^104^

Unlike the PROTACs presented by Zhou and Zheng, which recruit CRBN or VHL E3 ligases, in 2022, Chang et al.^105^ presented the first PROTAC targeting BCL‐XL by recruiting the MDM2 E3 ligase. There are several studies indicating that the choice of the recruited E3 ligase has a tremendous impact on the selectivity of the compound, as it can define a different band region of surface lysine of the target.^103^ Thus, three PROTACs that incorporate at one end the inhibitor navitoclax, and at the other end the MDM2 inhibitor, nutlin‐3, linked together by different types of linkers, were reported.^105^ Among them, PROTAC BMM4 (25) was the most promising as it demonstrated a potent and selective degradation activity against BCL‐XL in leukemic cells, accompanied by a significant stabilization of the tumor suppressor p53.^105^ When tested on AML cells that express WT p53, Western blot analysis demonstrated that PROTAC 25 degraded POI and increased p53 at 10 μM.^105^ Furthermore, the concomitant use of PROTAC BMM4 with venetoclax has been shown to result in a more pronounced cytotoxic effect, that is, after 48 h, 10 μM of PROTAC alone induced 26% apoptosis, whereas the concomitant use of PROTAC with the inhibitor induced 40% apoptosis.^105^

### 3.3 Bruton's tyrosine kinase

Belonging to the Tec family, BTK, is expressed in all hematopoietic cells, with the exception of T cells and mature plasma cells, and is involved in the maturation, function, and differentiation process of B‐cells, through its participation in the B‐cell receptor (BCR) signaling pathway.^106^ When an antigen stimulates the BCR, it induces the phosphorylation of BTK, and consequently its activation.^107^ Thus, BTK conducts several proliferative and prosurvival pathways, which lead, for example, to the induction of transcription of growth factors and antiapoptotic proteins, promoting cell proliferation and increasing cell survival capacity.^108^ It has been demonstrated that BCR signaling is constitutively active in CLL patients, leading to BTK being associated with the development of CLL, making it an important drug target in this type of leukemia very common in the Western world.^109^, ^110^

#### 3.3.1 Inhibitors of BTK

Currently, the use of SMIs has been the most common approach for targeting BTK.^111^, ^112^ Of particular note in clinical practice is the use of the irreversible inhibitor ibrutinib, which covalently binds to cysteine 481 of the BTK kinase domain. However, the occurrence of mutations that prevent the covalent bond of ibrutinib with BTK (e.g., substitution of cysteine 481 with serine—C481S), means that the inhibitor can only bind reversibly to the target, often leading to the occurrence of clinical relapses.^113^ Even so, it has been demonstrated that CLL cells require BTK signaling to survive, and in this sense, new therapies capable of remaining effective even in the presence of mutations could be an added value in the treatment of CLL.^108^

#### 3.3.2 PROTACs against BTK

The first report regarding a PROTAC targeting BTK was in 2018 when Gray's group demonstrated that it is possible to degrade this protein through a multikinase degrader (TL12‐186 (28)) (Table 4).^114^ Acting on this information, this same group designed a selective BTK‐based PROTAC DD‐04‐015 (29), using the inhibitor RN486 as TLM, which selectively degrades the target and reduces cell proliferation.^114^

**TABLE 4:** Chemical structure of PROTACs targeting BTK protein.

No.	Chemical structure	PROTAC information	References
28	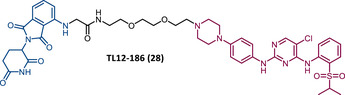	TLM: TL13‐87 ELM: Cereblon ligand (pomalidomide) DC_50_: NA	114
29	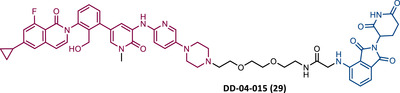	TLM: RN486 ELM: Cereblon ligand (pomalidomide) DC_50_: NA	114
30	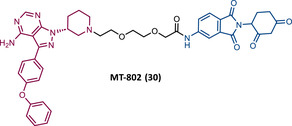	TLM: Ibrutinib ELM: Cereblon ligand (thalidomide derivative) DC_50 _= 9.1 nM (NAMALWA cells)	108
31	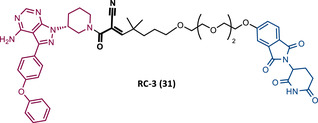	TLM: Ibrutinib ELM: Cereblon ligand (thalidomide derivative) DC_50_ = 6 nM (MINO cells)	115
32	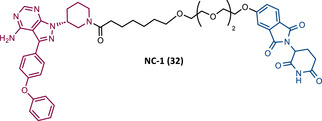	TLM: Ibrutinib ELM: Cereblon ligand (thalidomide derivative) DC_50 _= 2.2 nM (MINO cells)	115
33	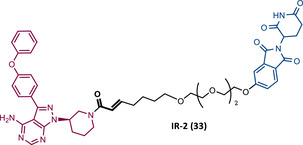	TLM: Ibrutinib ELM: Cereblon ligand (thalidomide derivative) DC_50_ = 1.9 nM (MINO cells)	115
34	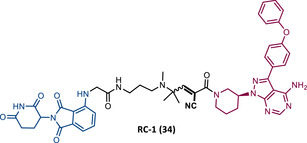	TLM: Ibrutinib ELM: Cereblon ligand (pomalidomide) DC_50_ = 6.6 nM (MOLM‐14 cells)	116
35	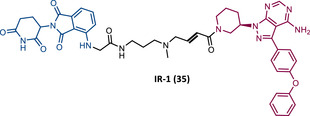	TLM: Ibrutinib ELM: Cereblon ligand (pomalidomide) DC_50_: NA	116
36	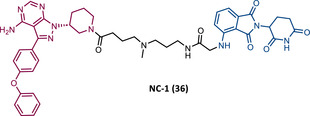	TLM: Ibrutinib ELM: Cereblon ligand (pomalidomide) DC_50_: NA	116

*Note*: 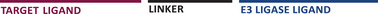

Abbreviations: ELM, E3 ligase moiety; NA, not applicable; TLM, target ligand moiety.

Although ibrutinib is unable to covalently bind to BTK in the presence of the C481S mutation, and consequently, is unable to inhibit this mutated target, the presence of the C481S mutation does not lead to the complete loss of the binding capacity of this inhibitor to the target.^108^ Even more, a PROTAC to promote the degradation of the target does not necessarily need to bind covalently to it, which means that the PROTAC is still able to bind noncovalently to the target, and thus promote its degradation.^108^

Based on this information, Crews’ group studied the possibility of a PROTAC targeting BTK, using an ibrutinib analogue as TLM, for its effectiveness in treating CLL with C418S BTK.^108^ Studies with the CRBN‐based PROTAC MT‐802 (30) demonstrated that this degrader was capable of degrading WT BTK with nanomolar potency (DC_50 _= 9.1 nM).^108^ Furthermore, it has also been shown to degrade BTK^C418S^, also with nanomolar potency, in CLL cells from relapsed patients.^108^ It should be noted that PROTAC MT‐802 was more selective for BTK than the parent inhibitor ibrutinib.^108^ Unfortunately, there are no in vivo studies with this degrader. However, this is an excellent example of how suboptimal TLM can be useful in the design of new PROTACs.^108^ From a SAR perspective, it is worth noting that this was one of the first reports of an CRBN‐based PROTACs in which changing the linker attachment point from C4 of the phthalimide ring to C5 demonstrated a significant increase in the potency of the degrader. This indicates that the ELM binding site has impact on the ease of PROTAC in forming a stable ternary complex, and therefore its ability to promote target degradation.^108^

Among the numerous advantages presented by PROTACs, one of the highlights is their ability to promote sub‐stoichiometric degradation due to their catalytic mechanism of action. However, some studies demonstrate that the introduction of irreversible TLMs reduces the potency of the degrader, as they make PROTAC's catalytic activity impossible.^115^, ^116^ So, from a theoretical viewpoint, the design of reversible covalent (RC) PROTACs may allow maintaining the catalytic activity of the degrader, as well as preserving the beneficial characteristics originating from a covalent bond (e.g., enhanced potency, selectivity, long duration of action).^115^, ^116^

In order to test this hypothesis, several groups have addressed the impact that the reversible or irreversible nature of PROTAC may have on its activity.^115^, ^116^

In 2020, the London's group designed a set of new cyanoacrylamide‐based RC PROTACs targeting BTK protein, which at the time was already known to be a good target for noncovalent (NC) PROTACs.^115^ In this way, NC, irreversible covalent (IR), and RC PROTACs were synthesized, some of which were active in the primary cells from CLL patients.^115^ Studies done with irreversible PROTACs demonstrated that part of the induced degradation occurs before the formation of the covalent bond, while in RCs it occurs mainly through covalent engagement.^115^ Among the PROTACs studied, PROTACs RC‐3 (31) and NC‐1 (32) stand out.^115^ Although both had similar cellular permeability and reversibly bound to BTK, PROTAC NC‐1 proved to be the most active (DC_50 _= 2.2 nM, *D*_max _= 97%) compared with RC‐3 (DC_50 _= 6 nM, *D*_max _= 85%), and even compared with IR‐2 (33) (DC_50 _= 1.9 nM, *D*_max _= 88%).^115^ On the other hand, the RC‐3 had enhanced selectivity, since the addition of cyanoacrylamide with the geminal dimethyl group reduced the reversible binding capacity of TLM, maintaining the covalent bond, which lead to PROTAC binding to less NC off‐targets.^115^ However, from this study, it is not possible to prove that RC PROTACs are more advantageous than NC or irreversible PROTACs, as they have demonstrated to be less potent.

Wang's group also dedicated themselves to studying the impact of reversibility and the type of bond formed between the target and the TLM on the performance of PROTACs.^116^ Comparing the RC BTK PROTAC from London's group (RC‐3), with the RC PROTAC (RC‐1 (34)) from Wang's group, they are both cyanoacrylamide‐based RC PROTACs, however, the latter has the dimethyl group in the γ‐position of the cyanoacrylamide group.^115^, ^116^ Wang's studies confirm that cyanoacrylamide‐based RC PROTACs exhibit enhanced drug accumulation and target engagement (TE) in cells.^116^ The cyanoacrylamide groups react reversibly and with rapid kinetics with thiol groups. This rapid and reversible reaction with intracellular glutathione, which functions as the sink to trap RC‐1 in cells, or their binding to membrane cysteine residues that facilitate its membrane permeation, favors the intracellular accumulation of the PROTAC.^116^

When the CRBN‐based PROTACs synthesized by Wang et al.^116^ (RC‐1, IR‐1 (35), and NC‐1 (36)) were tested in MOLM‐14 cells, it was found that IR‐1 led to poor degradation of BTK, and that RC‐1 was the most potent of all at low concentrations (DC_50 _= 6.6 nM), proving to be one of the most powerful BTK‐based PROTACs reported so far.^116^ However, it should be noted that PROTAC RC‐1 and its homolog RC‐1‐methyl (incapable of inducing BTK degradation) had similar IC_50_ in the inhibition of MOLM‐14 cell proliferation (0.31 vs. 0.21 μM), which suggested that the cell growth inhibitory effect is due to the inhibition of BTK rather than its degradation.^116^ Thus, the high potency of PROTAC RC‐1 is most likely the result of the combination of its high intracellular accumulation, which allows a greater TE, with its inhibitory and degradative effect on BTK. PROTAC RC‐1 induced the degradation of BTK with equal potency and in a dose‐dependent manner, regardless of its mutation status, while PROTAC RC‐3 from the London group only degraded WT BTK.^115^, ^116^ Furthermore, RC‐1 presented greater selectivity for the target, which is in line with what was reported by the previous group.^116^ When tested in vivo, RC‐1 had a half‐life time of 3.4 h, with a maximum concentration of 20 μM. RC‐1‐treated mice showed half the BTK levels compared with the control group.^116^

In summary, PROTACs with RC cyano‐acrylamide moieties represent a strategy that generally improves the intracellular accumulation of the degrader. This addresses one of the main problems associated with PROTACs, as their high molecular weight, hinders their passage into the target cell.

In 2021, the same London's group published an article that resulted from the study of their previous most potent PROTAC, NC‐1, in CLL cells. The aim was to evaluate the impact of this degrader on the BCR signaling pathway, migration, and apoptosis induced by BTK degradation.^117^ Studies on CLL cells considered that NC‐1 is the most potent of the PROTACs previously presented by London's group, largely due to the fact that by not binding covalently to BTK, allowed it to bind and dissociate quickly.^117^ Furthermore, the forced degradation of BTK and phosphorylated BTK by NC‐1 resulted in a partial abolition of the activation of the BCR signaling pathway, greater than that achieved with ibrutinib.^117^

When CLL cells carrying mutations in the BTK gene (C481S or C481F mutation) were subjected to treatment with NC‐1, there was a decrease in BTK, as well as a reduction in the activation of the respective downstream elements (Akt and ERK).^117^ Treatment with ibrutinib under the same conditions proved to be ineffective.^117^ When studying the effects of inducing BTK degradation by NC‐1, it was found that this PROTAC induced mild cell apoptosis (10% higher in NC‐1 treated cells after 18 h), in addition to reducing cell migration with 100 nM for 18 h, with 100 nM for 48 h it overcomes the antiapoptotic effect of mesenchymal stromal cells.^117^

### 3.4 BET family

The bromodomain and extraterminal (BET) protein family has been extensively studied over the years, since these bromodomain‐containing proteins, known as epigenetic readers, recognize acetylated proteins (such as histones or TFs), and therefore play a role important in the regulation of genetic transcription.^118^

Among the various members of the BET family, there is a BRD4, which is a nuclear protein involved in the organization and regulation of genetic transcription of relevant genes (e.g., c‐Myc, BCL‐XL, BCL‐2).^118^, ^119^ Although its role is not yet completely clear in cancer development, since recent evidence indicates that BRD4 relevance in cancer goes beyond its role in transcription regulation, it is known that the BRD4 protein is often found overexpressed and abnormally activated in several types of cancer, including AML, ALL, and CML, and has therefore been extensively studied as a promising target involved in tumorigenesis and tumor progression.^120^, ^121^

#### 3.4.1 Inhibitors of BET proteins

Currently, there are already some small molecule BET inhibitors with considerable therapeutic effects, such as JQ1 (the first BET inhibitor), OTX015, or ABBV‐744 (phase I for the treatment of AML).^118^ However, all BET protein‐targeted drugs are in preclinical and clinical studies, given the difficulties presented, such as short half‐life times, protein accumulation, or adverse effects, such as thrombocytopenia, fatigue, or gastrointestinal problems.^118^, ^122^

#### 3.4.2 PROTACs against BRD4

**FIGURE 6:**
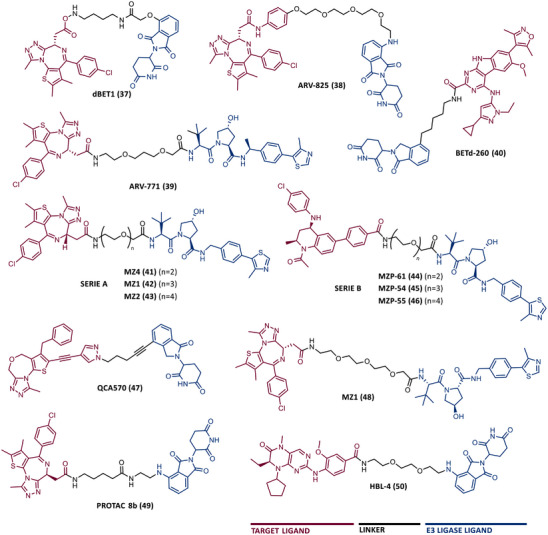
Chemical structure of PROTACs targeting BET family. Analysis of the structure of PROTACs targeting the BRD4 protein against leukemia, where the blue part represents the E3 ligase ligand, and the purple part represents the target ligand, joined together through a linker.

In 2015, 5 years after Bradner's group presented the first BET inhibitor, JQ1, this same group published a powerful CRBN‐based PROTAC targeting BRD4, designated dBET1 (37) (Figure 6).^123^, ^124^ This PROTAC results from the connection of the carboxyl group on JQ1 with the aryl ring of thalidomide by an alkyl‐based linker.^124^ In in vitro studies with human AML cell line (MV4;11) it was observed that dBET1 causes a marked reduction in BRD4 levels (>85%) at concentrations in the nanomolar range (DC_50 _= 430 nM).^124^ The induction of BRD4 degradation by this degrader resulted in a potent antiproliferative effect, superior to that obtained with the JQ1 inhibitor alone.^124^ In vivo studies demonstrated that dBET1 has a superior antileukemic effect than JQ1.^124^ Administration of dBET1 reduced tumor progression in murine xenograft model of human AML cells, accompanied by degradation of BRD4 and downregulation of c‐Myc.^124^

In 2022, a more comprehensive study of the effects of the dBET1 degrader on AML was published by Hu's group, based on the complexity and heterogeneity of this type of leukemia.^125^ Given that BRD4 overexpression is associated with a worse prognosis of AML, dBET1 was tested in several AML cell lines representative of the different AML subtypes, against which it was shown to be strongly cytotoxic.^125^ From the analysis of the impact of PROTAC on BRD4 downstream signaling, a reduction in c‐Myc expression was observed.^125^ Consequently, in all cell lines tested, a marked antiproliferative effect by dBET1 was observed, blocking the cell cycle and activating cell apoptosis.^125^ In summary, these studies demonstrated that PROTAC dBET1 had a comprehensive antileukemic effect for the various subtypes of AML, presenting potential benefits for the various AML patients.

Also, in 2015, Crews’ laboratory designed a CRBN‐based PROTAC called ARV‐825 (38), capable of binding to BRD4 by incorporating the OTX015 inhibitor, in turn linked to pomalidomide through a PEG‐based linker.^126^ Although it was not initially studied in leukemic cell lines, an article published in 2017 reported the study of ARV‐825 in AML cells.^127^ According to this study, the degrader significantly degraded BRD4 by more than 90%, quickly and with a long‐lasting effect, strongly inducing cellular apoptosis, both in cultured cells and in patient‐derived AML cells.^127^ In turn, the OTX015 inhibitor tested alone caused an intracellular accumulation of BRD4.^127^ Studies of the impact of ARV‐825 on the BRD4 signaling pathway demonstrated that PROTAC caused a more pronounced reduction in the levels of c‐Myc, CDK4/6, STAT3/5, among others, than OTX015.^127^ Consequently, BRD4 degradation was more lethal than its inhibition for AML cells.^127^ In this study, a new BET‐PROTAC called ARV‐771 (39) was also analyzed.^127^ This degrader was reported in 2016, and unlike ARV‐825, it recruits the VHL E3 ligase.^127^ When tested in vivo, in mice engrafted with luciferase transduced AML cells, the BET‐degrader ARV‐771 was more potent in reducing the leukemic load, increasing the survival rate, thus overcoming the effects observed with the use of the OTX015 inhibitor.^127^

In 2021, Wu et al.^128^ studied PROTAC ARV‐825 with the aim of evaluating its potential in the treatment of T‐ALL, since BRD4 overexpression is associated with poor prognosis in T‐ALL patients. Through cell viability studies in several T‐ALL cell lines, this group concluded that PROTAC ARV‐825 presents superior cytotoxicity than the JQ1 and OTX015 inhibitors, and even superior to the dBET1 degrader, in all cell lines tested.^128^ In more detail, PROTAC ARV‐825 generated a complete CRBN‐dependent degradation of BRD4 with DC_50_ values around 5 nM in MOLT‐4 and Jurkat cells.^128^ Consequently, the induction of this degradation translates into a decrease in c‐Myc levels, blocking the cell cycle and leading to cell death by apoptosis.^128^ In primary T‐ALL cells it also had a potent antitumor effect, due to the depletion of BRD4. In T‐ALL xenograft model, a considerable reduction in leukemic burden was observed after treatment with ARV‐825, which is consistent with what was observed in in vitro tests.^128^

In 2017, Wang's laboratory reported a series of new CRBN‐based PROTACs that feature as TLM new azocarbazole‐based BET inhibitors.^129^ Through linker optimization, powerful BET degraders were obtained, with emphasis on compound BETd‐260 (40) capable of degrading BRD4 with picomolar potency (30 pM) in RS4;11 leukemia cells.^129^ This degrader inhibited cell proliferation in RS4;11 and MOLM‐13 AML lines with IC_50_ values of 51 pM and 2.3 nM, respectively, being 1000 times more potent than the isolated warhead relative in reducing c‐Myc.^129^ Furthermore, it induced rapid tumor regression (>90%) in RS4;11 xenograft tumors, without significant adverse effects in mice, with a single administration capable of causing complete degradation of BRD4 for more than a day, with induction of cell apoptosis.^129^

With the aim of evaluating the impact that the target warhead and the linker have on PROTAC performance, Ciulli's group, in 2018, designed two series of VHL‐based PROTACs.^130^ Both series feature the VHL ligand VH032 as ELM, and PEG‐based linkers of different lengths.^130^ In series A (41‐43), TLM is a triazolodiazepine inhibitor (JQ1), in series B (44–46), TLM is a tetrahydroquinoline inhibitor (I‐BET726), more potent than the previous one.^130^ From the SAR studies it was found that the tetrahydroquinoline‐based series presented negative cooperativity in the formation of the ternary complex, compared with the triazolodiazepine series, which presented positive cooperation, being generally more potent degraders.^130^ These studies demonstrated that the incorporation of more potent TLM does not directly translate into a PROTAC with better degradation activity, which is truly impacted by the ease of formation of the stable ternary complex. The length of the linker was shown to impact the cellular activity of the degrader (PEG‐3 > PEG‐4 > > PEG‐2), suggesting the existence of a “sweet spot” for the length of the linker depending on the ELM‐TLM pair.^130^

Through structure‐based design, Wang's group designed a new class of BET inhibitors, the 1,4‐oxazepines, taking as an example the lead compound QCA276 (47).^131^ Subsequently, this group incorporated this potent inhibitor into CRBN‐based PROTACs, obtaining a potent degrader, QCA570 (35), which degraded BRD4 and inhibited cell growth in AML cell lines with picomolar potency (IC_50_ values between 8.3 and 32 pM), being 1000 times more potent than dBET1 in all cell lines tested.^131^ In vivo, the degrader reduced BRD4 levels, with a consequent reduction in c‐Myc levels, inducing a strong activation of cell apoptosis by cleavage of PARP.^131^ In both MV4;11 and RS4;11 acute leukemia xenograft models, PROTAC QCA570 induced complete and long‐lasting tumor regression, without causing toxicity in mice.^131^

In 2022, Ma et al.^132^ reported a new VHL‐based PROTAC called MZ1 (48). To build this PROTAC targeting BRD4, the JQ1 inhibitor connected via a PEG‐based linker to VHL‐1 was incorporated.^132^ By promoting the degradation of BRD4, both c‐Myc and ANP32B were downregulated by this degrader, resulting in cytotoxic effects (inhibition of cell proliferation, induction of apoptosis, and cell cycle arrest at G1) at low concentrations, in several representative AML cell lines of different sub‐molecular types.^132^ Investigation of the activity of MZ1 in vivo demonstrated that the group treated with the degrader showed a significant reduction in tumor burden compared with the control group, due to the induction of POI degradation.^132^

More recently, Zhu's group designed a set of new CRBN‐based PROTACs targeting BRD4.^133^ The lead compound was PROTAC 8b (49), which inhibited cell proliferation with IC_50_ values between 3 and 27 nM, obtained through the induction of BRD4 degradation in a dose‐ and time‐dependent manner.^133^ From SAR studies, it is possible to verify that the incorporation of a phenyl group as a binding vector between the linker and ELM, in the 4‐position, reduced the activity of PROTAC.^133^ On the other hand, if the vector is ethylenediamine, it generates a degrader (8b) that inhibits cell proliferation with IC_50_ values between 3 and 27 nM, obtained through the induction of BRD4 degradation in a dose‐ and time‐dependent manner.^133^ Replacing nitrogen with oxygen in the 4‐position leads to a reduction in PROTAC activity.^133^

#### 3.4.3 PROTACs against BRD4 and PLK1

Polo‐like kinase 1 (PLK1) protein is considered a relevant therapeutic target in AML, along with the BRD4 protein.^134^ PLK1 is involved in cell replication by performing functions in the M phase of the cell cycle, binding and phosphorylating a set of proteins that promote cell proliferation.^135^ However, it is frequently overexpressed in several diseases, including AML. Its inhibition through volasertib (BI6727) has been studied in AML, where it was discovered that in addition to inhibiting PLK1, this compound also inhibited BRD4, which was an advantage given that previous studies have shown that PLK1 inhibition synergized the effect of BRD4 inhibitors on AML.^134^, ^136^

Combining the potential that the dual inhibition of PLK1 and BRD4 has, with the advantages presented by PROTACs, in 2020, Xupeng et al. reported a pioneering dual PLK1 and BRD4 degrader designated HBL‐4 (50).^136^ Thus, the design of this degrader opened doors to the investigation of innovative single poly‐pharmacological targeting compounds. The HBL‐4 degrader resulted from the binding through the phenyl group of the volasertib inhibitor, by a PEG‐based linker, to a CRBN ligand (pomalidomide).^136^ Studies with PROTAC HBL‐4 demonstrated that it can potently inhibit cell proliferation in several AML cell lines, such as MOLM‐13 (IC_50_ = 6.21 nM), KG1 (IC_50_ = 6.94 nM), and MV4;11 (IC_50_ = 4.48 nM) cells.^136^ Degradation studies demonstrated that HBL‐4 rapidly and with nanomolar potency significantly degraded PLK1 in all AML cell lines tested (DC_50_ value between 10 and 20 nM).^136^ Interestingly, the degrader blocks MV4;11 in the G0/G1 phase of the cell cycle, whereas the volasertib inhibitor did so in the G2/M phase.^136^ Furthermore, in this same cell line, PROTAC reduced migration to a greater extent than the inhibitor.^136^ From the analysis of in vivo data, it is possible to infer that HBL‐4 degrader had great antitumor effects in MV4‐11 tumor xenograft model, through the degradation of PLK1 and BRD4, in a well‐tolerated way, surpassing the effects observed with the use of TLM inhibitor.^136^

### 3.5 CDK family

The CDKs family includes more than 21 enzymes, classified as serine‐threonine (S/T) kinases, essential in the vital and housekeeping activities, such as the cell cycle (CDK1, 2, 4, 6, and 11) or gene expression regulation (CDK7, 8, 9, and 11).^63^, ^137^ The activity of these kinases is regulated by their binding to cyclins, hence their name. This binding results in the phosphorylation of a set of substrates.^63^, ^138^

CDK6 is essential in cell cycle regulation because it is involved in controlling the G1/S transition.^63^ By associating with D‐type cyclins, CDK6 is activated, and phosphorylates the tumor suppressor retinoblastoma (Rb), which once phosphorylated, leads to the repression of E2F TFs.^139^ Consequently, the triggering of gene expression necessary for entry into the S phase of the cell cycle does not occur.^140^ It is also known that CDK6, through mechanisms independent of its kinase activity (scaffolding functions), is involved in the regulation of metabolism and genetic transcription, mediating growth‐promoting functions.^141^, ^142^ Often, CDK6 is hyperactivated or overexpressed, resulting in uncontrolled cell proliferation, which is why it is considered a hallmark of cancer.^143^ In leukemia, CDK6 plays a prominent role since it is associated with the uncontrolled LSCs proliferation, leading to the development of AML and CML.^144^, ^145^ Furthermore, mixed‐lineage leukemia (MLL) and Ph‐positive ALL (ALL‐Ph^+^) cells are also dependent on CDK6 for their survival.^146^, ^147^

#### 3.5.1 Inhibitors of CDK6

ATP‐competitive CDK6 inhibitors (CDK6i) such as palbociclib, ribociclib, or amebaciclib have been studied for the treatment of various types of cancers.^140^ However, given the high level of homology between CDK4 and CDK6 in their ATP binding pockets (greater than 90% amino acid sequence identity), inhibitors are not selective for CDK6.^138^ Treatments with these CDK6i are closely associated with the occurrence of neutropenia, due to the inhibition of CDK4/6 in hematopoietic progenitors.^143^, ^144^, ^148^ Furthermore, the occurrence of resistance due to point mutations in CDK6, as well as these inhibitors only affect the kinase‐dependent functions of CDK6, are some of the shortcomings associated with CDK6i.^147^, ^149^

#### 3.5.2 PROTACs against CDK6

The construction of PROTACs targeting CDK6 is theoretically an asset, insofar as, by promoting its degradation they manage to disrupt both its catalytic and scaffolding functions.^61^

In a pioneering study carried out in 2018 by Gray's group, a CRBN‐dependent multikinase degrader based on a diaminopyrimidine scaffold, TL12‐186 (28), was reported.^114^ This PROTAC results from the conjunction of pomalidomide, with a PEG linker, to the TAE684 inhibitor, which is highly promiscuous, binding to several kinase enzymes, including BTK and CDK6.^114^ TL12‐186 was shown to be capable of degrading CDK6 for the first time, which opened the door to the development of new PROTACs selective for CDK6.^114^

In the same year, Brand et al.^138^ developed a selective homologous CDK6 degrader, designated BSJ‐03‐123 (BSJ) (51) (Table 5).^138^, ^150^ This PROTAC is the successor to compound YKL‐06‐102 (52), which links pomalidomide to solvent‐exposed piperazine on palbociclib via a 3‐PEG linker.^150^ While selectively degrading CDK6, YKL was found to destabilize the IKZF1/3 proteins.^138^, ^150^ In order to remove this unwanted activity, PROTAC BSJ was created, to which a phenoxyacetamide linker of equal length was added.^150^ This structural alteration allowed the BSJ degrader to promote a potent, rapid, and selective CRBN‐dependent degradation of CDK6, for example, immunoblot studies demonstrated a significant reduction in CDK6 levels when using concentrations from 50 nM of PROTAC BSJ‐03‐123.^138^ In vitro kinase assays demonstrated that although BSJ had equal affinity for CDK4 and CDK6, it only degrades the latter, hypothesizing the cause for the formation of a differential ternary complex, that is, it only forms a stable ternary complex with CDK6, promoting its degradation.^138^, ^150^ When tested in CDK6‐dependent AML cell lines, PROTAC BSJ caused a significant antiproliferative effect by inducing G1 cell‐cycle arrest, yet without an increase in the number of apoptotic cells.^138^, ^150^ Comparing CDK6 degradation with CDK4/6 inhibition, it was found that degradation does not have superior advantages, which could be related to the fact that AML cells are dependent only on the kinase function of CDK6.^138^, ^150^

**TABLE 5:** Chemical structure of PROTACs targeting CDK6 protein.

No.	Chemical structure	PROTAC information	References
51	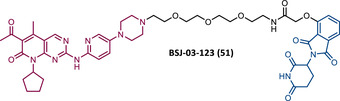	TLM: Palbociclib ELM: Cereblon ligand (thalidomide) DC_50_: NA	138
52	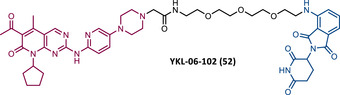	TLM: Palbociclib ELM: Cereblon ligand (pomalidomide) DC_50_: NA	150
53	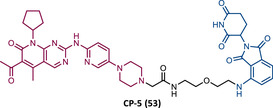	TLM: Palbociclib ELM: Cereblon ligand (pomalidomide) DC_50_ = 1.1 nM (U251 cells)	149
54	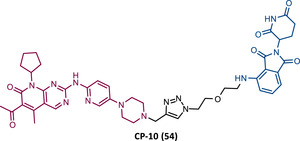	TLM: Palbociclib ELM: Cereblon ligand (pomalidomide) DC_50_ = 2.1 nM (U251 cells)	149
55	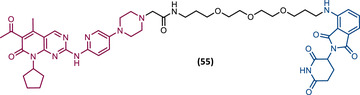	TLM: Palbociclib ELM: Cereblon ligand (thalidomide derivative) DC_50_: NA	137
56	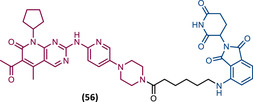	TLM: Palbociclib ELM: Cereblon ligand (thalidomide derivative) DC_50_: NA	151
57	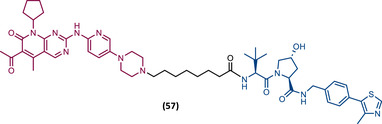	TLM: Palbociclib ELM: VHL ligand (VH032 ligand) DC_50_ = 6.6 nM	151
58	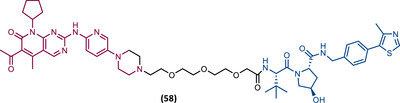	TLM: Palbociclib ELM: VHL ligand (VH032 ligand) DC_50_: NA	151
59	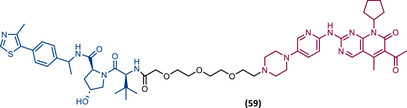	TLM: Palbociclib ELM: VHL ligand (VH032 ligand) DC_50_: NA	151
60	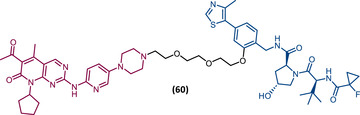	TLM: Palbociclib ELM: VHL ligand (VH032 ligand) DC_50_ = 5.1 nM	151
61	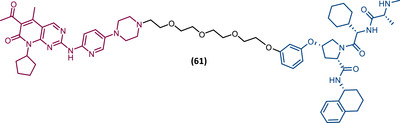	TLM: Palbociclib ELM: VHL ligand (VH032 ligand) DC_50_: NA	151
62	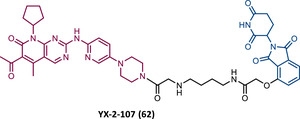	TLM: Palbociclib ELM: Cereblon ligand (thalidomide derivative) IC_50_ = 4.4 nM (BV173 cells)	153
63	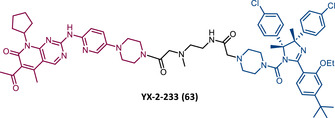	TLM: Palbociclib ELM: MDM2 ligand DC_50_: NA	153

*Note*: 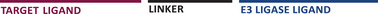

Abbreviations: ELM, E3 ligase moiety; MDM2, mouse double minute 2; NA, not applicable; TLM, target ligand moiety; VHL, Von Hippel‐Lindau.

The binding affinity to the target, the chosen TLM, ELM, and linker, as well as the spatial orientation acquired by all intervenient during the formation of the stable ternary complex by PROTAC have an impact on the performance of the degrader. In order to study these factors, Rao's group created a library of compounds intended for the degradation of CDK6 for the treatment of various hematological diseases, including leukemia.^149^ As TLM, one of the three US FDA‐approved CDK4/6 inhibitors (palbociclib, ribociclib, and abemaciclib) were chosen, and linked by the piperazine group to different types of linkers, to a ligand capable of recruiting one of the following E3 ligases—CRBN, VHL, IAP, or the MDM2.^149^ However, only CRBN‐based PROTACs were able to degrade the target, and among them, those presenting palbociclib as TLM were the best.^149^ From the analysis of the palbociclib‐pomalidomide PROTACs, it is possible to verify that the choice of the linker is not indifferent, with small linkers such as those of the CP‐5 (53) or CP‐10 (54) compounds conferring a greater degradation capacity, which could result from the fact that they allow preferable spatial positions to recruit CDK6 and CRBN.^149^ Although the variation of the linker‐attaching end at the palbociclib side did not produce great effects, on the pomalidomide side, the introduction of rigid groups (rigid alkyne group) compared with imino or methylene groups, with greater flexibility, reduced the potency of the degraders.^149^ Among the various PROTACs synthesized, the compound CP‐10 stands out, capable of selectively inducing the degradation mediated by the UPS of CDK6, in 72% at 10 nM and 89% at 100 nM, with complete degradation after 6 h and no significant off‐target effects.^149^ Moreover, this PROTAC was also able to efficiently degrade mutant forms of CDK6 (D163G or S178P mutations) in resistant cell lines, inhibiting cell proliferation.^149^ It should be noted that the present PROTAC, similarly to what has been observed, has a weaker affinity than isolated palbociclib for CDK6, demonstrating that a lower affinity is not an impediment to the action of PROTAC.^149^

Both BSJ and CP‐10 PROTACs are in line with what was demonstrated by PROTAC 55 developed by Natarajan's group in 2019.^137^, ^138^, ^149^ All these palbociclib‐based PROTACs were much more selective for CDK6 than the respective isolated palbociclib.^137^, ^138^, ^149^ This selectivity could be explained by a greater stability in the ternary complex with CDK6, or by the fact that CDK4 had fewer lysine residues (11 in CDK4 vs. 18 in CDK6) required for the binding of ubiquitin units.^137^, ^138^, ^149^ Another possibility to consider was the CDK4 deubiquitination rate.^137^, ^138^, ^149^ However, further studies are needed.

Contrary to what had been seen previously, in which only CRBN‐based PROTACs were capable of selectively promoting the degradation of CDK6, in 2020, two different research groups presented two libraries of new palbociclib‐based PROTACs that, when recruiting the VHL or IAP E3 ligases, could promote potent degradation of CDK6.^151^, ^152^ These novel PROTACs allowed to overcome the limitations associated with CRBN‐based PROTACs, such as the occurrence of resistance due to the inactivation of this E3 ligase, or the off‐target effects related to the degradation of the IKZF1/3 proteins.^151^, ^152^

Regarding the work developed by Steinebach et al.,^151^ new, potent, and long‐lasting palbociclib‐based PROTACs capable of degrading CDK6 by recruiting CRBN, VHL, and IAP E3 ligases were reported. The CRBN‐based PROTACs series resulted from linking pomalidomide with palbociclib via PEG and other alkylidene‐based linkers.^151^ Some of these PROTACs had a selectivity ratio similar to BSJ, however, some lead to the degradation of IKZF1.^151^ Of all, PROTAC 56 stands out, which when tested in Ba/F3 cells induced degradation of the target at concentrations as low as 10 nM. Under the same conditions, PROTAC BSJ shows an attenuation of its activity when tested at concentrations lower than 0.1 μM.^151^

The VHL‐addressing CDK6 PROTACs incorporate palbociclib linked to derivatives of the VHL ligand VH032, by various types of linkers, and are divided into two subseries.^151^ The “amide subseries” are compounds in which the bond between the linker and the exit vector of the VHL ligand forms an amide, in the “phenoxy subseries” the linker is linked to the phenol group.^151^ All PROTACs in the amide series have been shown to degrade CDK6. PROTAC 57 features an alkyl linker, degrading both CDK4 and CDK6.^151^ However, when replaced with a PEG linker similar to that of BSJ, the selectivity for CDK6 increases (PROTAC 58).^151^ By adding a methyl group to the VHL ligand, PROTAC 59 demonstrates selectivity and improved concentration‐ and time‐dependent degradation of CDK6, surpassing the results obtained by BSJ.^151^ In the phenoxy subseries, PROTAC 60 stands out, which in MM.1S cells, has a DC_50_ value for CDK6 of 5.1 nM and a *D*_max_ greater than 95% with a concentration of 100 nM. It should be noted that PROTACs 59 and 60 had a longer duration of action compared with BSJ.^151^ With BSJ, CDK6 protein levels began to recover after 24 h after treatment, whereas with PROTACs 59 and 60, after 96 h, a satisfactory reduction in CDK6 was still observable.^151^ This could be related to the chemical inactivation of the CRBN ligand. Furthermore, PROTAC 60 has been shown to reduce cell migration capacity (by about 30%) when tested in AML cell lines (e.g., MOLM‐13, HEL, KG‐1, K562) or ALL (Nalm‐6).^151^ This work also presented for the first time an IAP‐based PROTAC (PROTAC 61) capable of degrading CDK4 and CDK6 at concentrations as low as 0.1 μM.^151^ Regarding the MDM2‐based PROTACs, none of them showed activity, which may be a consequence of their low cellular permeability.^151^

In 2020, Dominici et al.^153^ presented for the first time a set of CDK6‐targeted PROTACs specifically intended for the treatment of ALL‐Ph^+^.^148^, ^153^ This type of leukemia results from constitutive tyrosine kinase activity of the BCR‐ABL p190 isoform or, more rarely, of p210.^148^ Today, the dependence of CDK6 expression on the growth of ALL‐Ph^+^ cells is known, which in turn is less related to CDK4 activity due to its cytoplasmic localization.^148^, ^153^ Thus, the use of CDK4/6i, such as palbociclib, was shown to suppress the growth of these cells, but relapsed due to the development of resistance occurring in more than 50% of patients.^148^, ^153^ Based on previous studies that proved that the silencing of CDK6 in ALL‐Ph^+^ was more effective than its inhibition by palbociclib, revealing the importance of the kinase‐independent and dependent mechanisms of CDK6, several CDK6‐selective PROTACs were designed.^148^, ^153^

Among them, CRBN‐recruiting PROTAC YX‐2‐107 (62) stands out, which potently inhibits CDK6 in vitro with a IC*_50_* value of 4.4 nM, lower than that of palbociclib (IC_50_*__* = 9.5 nM).^153^ PROTAC YX‐2‐107 when tested in ALL‐Ph^+^ cell lines (Ph1 BV173 and SUP‐B13 cells) selectively degraded CDK6 (with a degradation constant of ∼4 nM) and inhibited the G1‐S transition, FOXM1 expression, Rb phosphorylation, and cell proliferation.^148^, ^153^ On the other hand, isolated palbociclib induced the expression of both CDK4 and CDK6.^153^ When tested on CD34^+^ normal hematopoietic progenitors, despite inducing CDK6 degradation, no cytostatic effect was observed, given the possible greater dependence of these cells on CDK4 activity.^153^ Therefore, selective degraders for CDK6 have a less toxic potential than CDK4/6i, with a consequent reduction in the occurrence of neutropenia that is highly associated with the use of this type of inhibitor.^148^, ^153^ Moreover, the present degrader, in addition to not interfering with CDK4, has also been shown to not interfere with the expression of IKZF1/3 proteins.^148^, ^153^ In vivo studies have shown that PROTAC 62 has good metabolic stability, with a half‐life time of 35 min.^148^, ^153^ In mice injected with de novo or TKI‐resistant primary ALL‐Ph^+^, PROTAC YX‐2‐107 induced marked suppression of leukemia burden comparable or even superior to that achieved with palbociclib.^148^, ^153^ In normal mice, exposure for 10 consecutive days to a high dose of the degrader did not induce significant changes in normal hematopoietic progenitors and mature cells.^148^, ^153^ However, cessation of treatment with PROTAC or palbociclib quickly translates into leukemia regrowth.^148^, ^153^

Similar to what had been verified previously, Dominici et al.,^153^ demonstrated that the incorporation of a CDK4/6i in the design of a CRBN‐based PROTAC increases its selectivity for CDK6.^148^, ^153^ This selectivity of CRBN‐based PROTACs is explained by the new protein–protein contacts that are established during the formation of the ternary complex, favoring the binding of PROTAC to CDK6 and not to CDK4.^148^, ^153^ On the other hand, the first MDM2‐based PROTAC YX‐2‐233 (63) able to degrade CDK6, also degraded the CDK4 protein.^148^, ^153^ This suggests that the choice of E3 ligase recruited by PROTAC is critical for its selectivity, and in turn, this depends on the interactions that favor the assembly of the ternary complex.

### 3.6 FMS‐like tyrosine kinase 3

FLT‐3 is a tyrosine kinase receptor and is a well‐documented oncogenic driver in AML.^154^, ^155^ Between 20% and 30% of newly diagnosed patients with this acute leukemia have mutations in the FLT‐3 gene.^156^, ^157^ The occurrence of these mutations leads to a constitutive activation of this protein, involved in processes of proliferation, differentiation, and survival of blood cells.^156^, ^157^ For example, the internal tandem duplication (ITD) represents the most common type of FLT‐3 mutation (FLT‐3‐ITD; approximately 25% of all AML cases) and is a common driver mutation associated with high leukemic burden and poor prognosis in AML patients.^156^, ^157^

#### 3.6.1 Inhibitors of FLT‐3

The FLT‐3 inhibitors (FLT‐3i) are used in the treatment of AML.^156^ First‐generation of FLT‐3i are multikinase inhibitors (e.g., sunitinib), with low potency, with many off‐target effects, and with reduced specificity for FLT‐3.^156^, ^158^ On the other hand, second‐generation ones (e.g., quizartinib, gilteritinib) are much more specific for FLT‐3, and potent.^156^, ^158^ However, their benefits are limited, and there are several known resistance mechanisms that lead to the failure of these drugs.^155^, ^159^ Therefore, new alternatives are needed, and PROTACs could be one of them.

#### 3.6.2 PROTACs against FLT‐3

**FIGURE 7:**
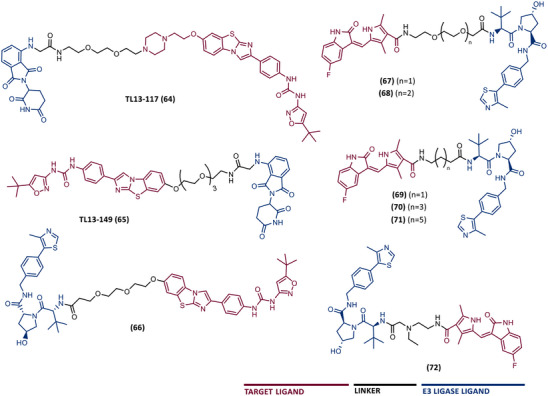
Chemical structure of PROTACs targeting FLT‐3. Analysis of the structure of PROTACs targeting the FLT‐3 protein against leukemia, where the blue part represents the E3 ligase ligand, and the purple part represents the target ligand, joined together through a linker.

Based on preliminary studies with the CRBN‐dependent multikinase degrader, TL12‐186 (28), Gray's group, in 2018, successfully created the first two CRBN‐based PROTACs selective for FLT‐3, PROTAC TL13‐117 (64) and TL13‐149 (65) (Figure 7).^114^ These degraders incorporate a clinical‐stage ATP‐competitive FLT‐3i, AC220, a PEG‐based linker, and a CRBN ligand.^114^ When tested in AML cell lines (MOLM‐14 cells) both PROTACs were shown to reduce target levels (with a maximum of efficient degradation around 10−100 nM), while AC220 inhibitor alone induced an increase in its expression.^114^ However, the action of PROTACs did not translate into an improved antiproliferative effect.^114^ Dose–response studies have also demonstrated the occurrence of the Hook effect at high doses of TL13‐177, that is, high doses of PROTAC favor the formation of dimers (POI–PROTAC or PROTAC––E3 ligase) instead of the desirable ternary complex (POI–PROTAC–E3 ligase) to induce protein degradation.^114^

In 2018, a novel PROTAC targeting FLT‐3 was reported, which resulted from the link between a quizartinib derivative (AC22013), whose morpholine group has been replaced by a PEG‐based linker, connected to a VHL ligand.^155^ Kinase activity assays demonstrated that PROTAC 66 inhibits both WT FLT‐3 (36 nM) and FLT‐3‐ITD (43 nM), although this inhibition is lower than that obtained with quizartinib (7.4 and 10 nM, respectively).^155^ Cellular proliferation assays in leukemic cell lines (MV4;11 and MOLM‐14 cells) demonstrated that the degradation of FLT‐3 induced by PROTAC exceeds the results obtained with quizartinib, suggesting that the degradation of this target is important for increasing the antiproliferative activity.^155^ Indeed, PROTAC 66 was over 3.5‐fold more potent than warhead alone (IC_50_ value of 0.6 vs. 1.87 nM, respectively).^155^ Furthermore, when studying the activity of this degrader in vivo, PROTAC degraded the FLT‐3‐ITD mutant with nanomolar potency, causing a 60% decrease in FLT‐3 levels compared with the control level.^155^ This enhanced antiproliferative activity of PROTAC 66 compared with the isolated inhibitor could be explained by the degradation of FLT‐3‐ITD mutant accelerating the activation of apoptosis in leukemic cells, which could be related to the nonkinase roles of FLT‐3.^155^

In 2022, Sang's group developed a series of new VHL‐based PROTACs targeting FLT‐3.^159^ From a structural viewpoint, the 12 PROTACs presented the result from the connection of a VHL ligand to the multitarget inhibitor, sunitinib, through various types of linkers (e.g., PEG, alkyl, alkylamine, piperazine).^159^ All PROTACs were tested on two leukemia cell lines (K562 and HL‐60), and based on their antiproliferative activity, it was found that PROTACs with PEG‐based linkers (67 and 68) were more active than those with alkyl‐based linkers (69, 70, and 71).^159^ In fact, PROTAC 67 is the one with the best antiproliferative activity, even surpassing the inhibitor alone, against HL‐60 cells (IC_50 _= 2.9 μM), in which 10 μM caused a complete degradation of the POI after 48 h.^159^ In the K562 cells, PROTAC 72 stands out with an IC_50_ value of 8.4 μM, unfortunately, higher than that obtained with sunitinib alone (IC_50 _= 5.8 μM).^159^ Also, it should be noted that both PROTACs 67 and 72 induced UPS degradation of FLT‐3 in K562 cells.^159^ When tested in a normal human cell line (NCM460) both PROTACs 67 and 72 showed reduced cytotoxicity (IC_50 _> 100 μM) compared with the tested tumor cells.^159^

### 3.7 MDM2

As previously mentioned, the MDM2 enzyme is an E3 ligase recruited by PROTACs to promote the forced degradation of certain oncoproteins.^160^, ^161^ However, its own degradation may bring advantages in the treatment of certain types of cancer.

Among the various substrates of MDM2, the tumor suppressor p53, commonly known as the guardian of the genome, stands out. Under normal conditions, the p53 protein is expressed at low levels due to its interaction with its negative endogenous regulator, MDM2.^162^ In other words, MDM2 is capable of biding to the N‐transactivation domain of p53, preventing p53 from binding to DNA.^163^ MDM2 further catalyzes the transfer of ubiquitin units to p53, which is subsequently recognized and degraded by the 26S proteasome.^162^ Furthermore, MDM2 promotes the export of p53 from the nucleus to the cytoplasm.^164^ Therefore, this E3 ligase is capable of preventing the transcriptional activity of p53 in three different ways. However, in situations of cellular stress (e.g., osmotic shock, hypoxia, DNA damage) p53 is stabilized and tetramerizes (which prevents its binding to MDM2), migrates to the nucleus where it binds to key genes of DNA molecules and promotes gene transcription responsible for regulating processes, such as the cell cycle, DNA repair, senescence, or, in more severe cases, cell apoptosis.^162^ In fact, more than half of cancers have mutations or deletions of the TP53 gene.^165^ For example, less than a quarter of AML cases have mutations or deletions of this gene.^166^ In TP53 WT leukemia cells, MDM2 is frequently found overexpressed, which results in an increased rate of degradation of the tumor suppressor and culminates in an impairment of its anticancer functions.^167^, ^168^

#### 3.7.1 Inhibitors of MDM2

As MDM2 is classified as an oncogenic E3 ligase, over the last few years different types of inhibitors have been developed, with emphasis on nutlins—cis‐imidazoline compounds (e.g., nutlin‐3 or idasanutlin)—which mimic the interaction between p53 and MDM2.^169^ By inhibiting this E3 ligase, they cause an increase in intracellular levels of the p53 suppressor.^161^ Nutlins have been the subject of clinical studies in patients with AML and myeloproliferative neoplasms.^168^ However, the p53‐MDM2 disruption caused by SMIs, with the activation of p53, results in a negative feedback mechanism, where there is an increase in the transcription of the MDM2 gene, which consequently leads to greater degradation of p53, and thus to a reduction in inhibitor activity.^161^ Furthermore, the use of these inhibitors is often associated with hematological and related toxicities (e.g., neutropenia or bone marrow failure) or the emergence of resistance.^164^

#### 3.7.2 PROTACs against MDM2

**FIGURE 8:**
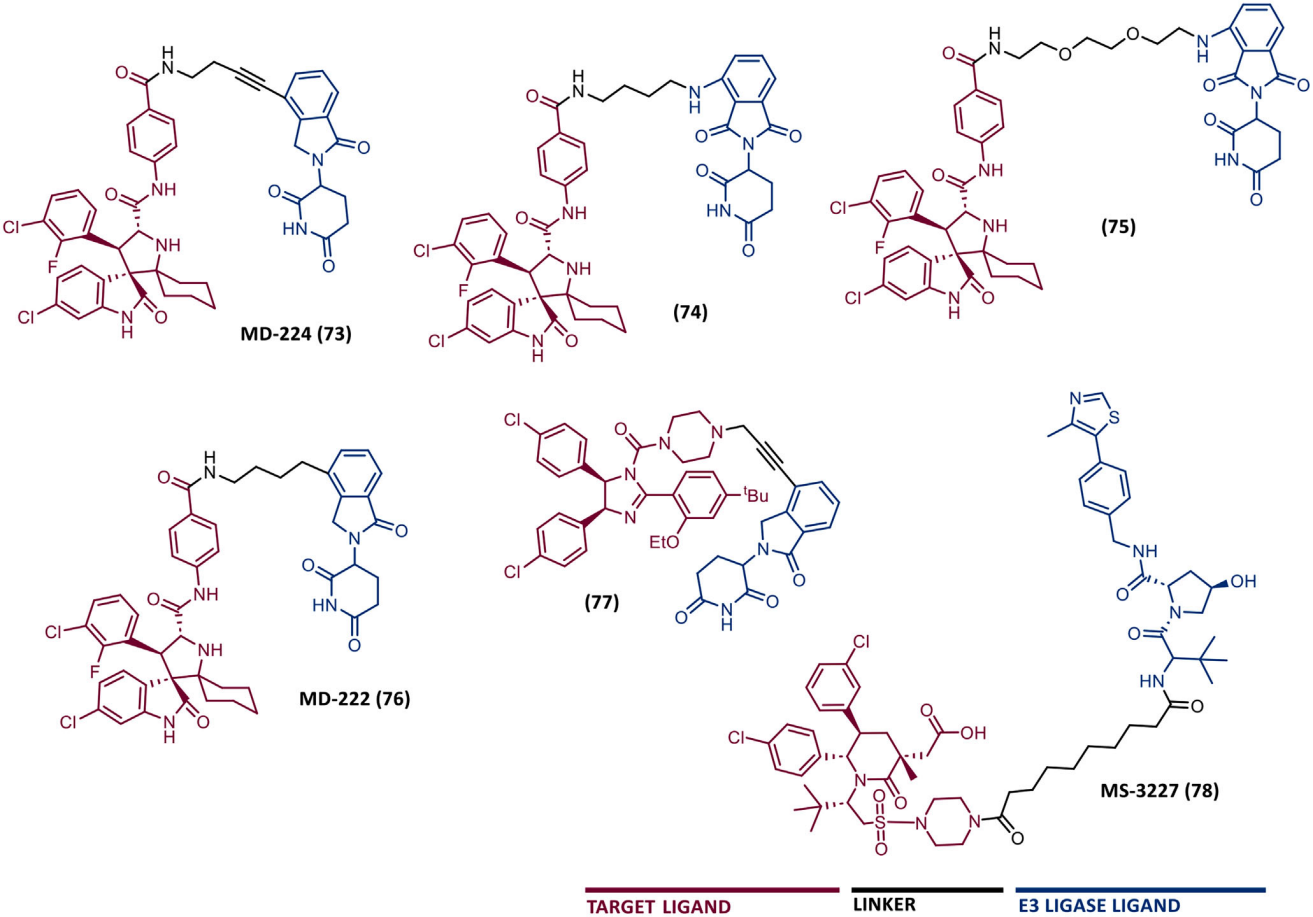
Chemical structure of PROTACs targeting MDM2. Analysis of the structure of PROTACs targeting the MDM2 protein against leukemia, where the blue part represents the E3 ligase ligand, and the purple part represents the target ligand, joined together through a linker.

In 2018, Wang's group at The Rogel Cancer Center discovered the first‐in‐class CRBN‐based PROTAC targeting the MDM2 ligase (MD‐224 (73)), which is capable of inducing with nanomolar potency rapid degradation of MDM2 in leukemic cells (Figure 8).^164^, ^165^ To construct the PROTACs, this group used a potent spirooxindole MDM2 inhibitor previously developed by them, MI‐1061 (Ki = 0.16 nM), in which the carboxylic acid group was converted into a methyl amide, giving rise to the inhibitor MI‐1242 (Ki = 2.7 nM), equally potent (IC_50 _= 89 nM) and which appears to present improved cellular permeability.^164^, ^165^ Linkers already reported in the literature were coupled to the inhibitor MI‐1242, and the CRBN ligands thalidomide and lenalidomide were chosen as ELM.^164^, ^165^

As expected, when the inhibitors MI‐1061 and MI‐1242 were tested in RS4;11 leukemia cells carrying WT p53, both led to an increase in MDM2 and p53 levels in a dose‐dependent manner.^164^, ^165^ On the contrary, PROTAC 74 and 75 were around 10 times more potent than the inhibitors, strongly preventing cell growth (IC_50_ values of 10 or 7 nM, respectively), and were able to increase p53 levels by reducing MDM2 levels through its degradation, even at concentrations as low as 3 nM.^164^, ^165^

It is extensively reported in the literature that aspects such as the length and composition of the linker have an impact on the performance of the degrader. Modifications in the linkers of PROTACs 74 and 75 demonstrated that linkers that are too short or too long reduce the activity of the compounds.^164^, ^165^ Furthermore, modifications of the ELM of the degrader molecule can also impact the activity of the compound.^164^, ^165^ For example, the change of the carbonyl group in pomalidomide in PROTAC 74, by a CH_2_ group (lenalidomide), associated with the replacement of the NH linking group by a methyl group, generated PROTAC MD‐222 (76), twice as potent (IC_50 _= 5 nM).^165^ In turn, replacing the NH linking group of MD‐222 with an alkyne group (linker rigidification) generated the powerful MD‐224 degrader (IC_50 _= 1.5 nM).^165^ Replacing the ELM of a CRBN ligand with a VHL ligand generated PROTACs with less capacity to reduce cell growth, presenting micromolar potency.^164^, ^165^ When tested on both RS4;11 and MV4;11 leukemia cell lines with WT p53, PROTACs MD‐222 and MD‐224 rapidly induced CRBN‐dependent degradation of MDM2 at concentrations as low as 1 nM, with MD‐224 the most powerful of all.^164^, ^165^ This degradation resulted in a powerful increase in the levels of the tumor suppressor p53, as well as in the levels of the PUMA gene (a potent proapoptotic gene), which results in the inhibition of cell proliferation and the activation of apoptosis (10–100 times more potent than inhibitors).^164^, ^165^ In vivo studies with PROTAC MD‐224 in mice bearing the RS4;11 xenograft tumor demonstrated that a single intravenous dose of the degrader induces the degradation of MDM2 and the stabilization of p53, with an effect persisting for more than 24 h.^164^, ^165^ Therefore, the authors stated that the present compound is capable of achieving efficient long‐lasting tumor regression, surpassing the results obtained with the inhibitor MI‐1061.^164^, ^165^

In 2019, Wang et al.^170^ developed PROTAC 77, a new CRBN‐based PROTAC targeting MDM2, which links through a single acetylene and one methylene unit the benzene ring of lenalidomide with the piperazine group of the analogue of the nutlin‐based inhibitor RG7112 (ligand (4S, 5R)−1). When tested in RS4;11 cell line, degrader 77 was the most potent compound, potently inhibiting cell growth (IC_50 _= 3.2 nM), surpassing the effects observed by the corresponding racemic compound (IC_50_ = 7.2 nM), and both being 1000 times more potent than inhibitor 1 alone, highlighting the advantage that PROTAC's catalytic mechanism of action (event‐driven mechanism) presents against occupancy‐driven inhibition.^170^ PROTAC 77 has a *D*_max_ value of 90% at 100 nM, and a DC_50_ value of 23 nM, being a potent MDM2 degrader, with the ability to increase the levels of the tumor suppressor p53.^170^ It should be noted that 100 nM of PROTAC 77 activates the apoptosis pathways, which culminates in the reduction of the proliferative activity of leukemic cells.^170^

All previously reported MDM2‐based PROTACs recruit the CRBN E3 ligase, and it was even reported by Wang's group that VHL‐based PROTACs targeting MDM2 would be poorly active.^168^, ^170^ However, in 2022, Marcellino et al.^168^ noted that in AML cells there is a relatively increased expression of the VHL E3 ligase, and therefore, the design of MDM2‐based PROTACs recruiting VHL could allow obtaining more selective compounds against leukemic cells. For the design of a library of VHL‐based PROTACs, this group linked the piperazine sulfonyl group of an analogue of the MDM2 inhibitor (AMG‐232), to a VHL ligand, through different linkers composed of alkylene or PEG.^168^ Based on SAR studies in human leukemia cell lines, PROTAC MS3227 (78), with an 8‐carbon linker, was identified as a potent MDM2 degrader.^168^ And when compared with the inhibitor AMG‐232, the degrader induced a greater increase in the levels of the p53 suppressor at similar concentrations.^168^ When tested on MOLM‐13 cells (AML), with a view to evaluating the ability to prevent cell proliferation, PROTAC MS3227 (IC_50 _= ∼50 nM) surpassed the effect caused by AMG‐232 (IC_50 _= ∼250 nM), also observed when tested on MOLT‐4 (ALL) leukemia cells.^168^ It should be noted that the effects presented by MS3227 are dependent on VHL expression and TP53 mutational status.^168^ When tested on primary patient AML cells, PROTAC MS3227 strongly induced apoptosis and cell death at 250−500 nM, an effect equal to or even greater than that induced by AMG‐232.^168^ It should be noted that the degrader is not active on AML cells with TP53 deletion, however, cells with mutational profiles and cytogenic abnormalities are sensitive to the effect of the degrader. Synergism studies demonstrated that PROTAC MS3227 can be combined with conventional AML therapeutics.^168^ For example, it has been studied that activation of the p53 signaling pathway by the degrader reduces the levels of MCL‐1, a prosurvivable factor that is responsible for the survival of leukemic cells and mechanisms of resistance to venetoclax (BCL‐2 inhibitor), resulting in an antileukemic effect appreciably higher.^168^

### 3.8 STAT family

The STAT family is made up of seven members, which share the highly conserved Src homology 2 (SH2) domain.^171^ Among the various members, STAT3 and STAT5 (which has two isoforms, called STAT5A and STAT5B) were the proteins chosen as targets for the development of PROTACs for the treatment of leukemia.^172^ Under normal conditions, STAT3 is activated in response to various cytokines or growth factors, regulating a set of different genes involved in cell survival and proliferation, as well as in metastasis, immune evasion or drug resistance events.^171^, ^173^ Although the central pathogenesis of CML is related to the BCR‐ABL fusion protein, it is known that this oncoprotein leads to the constitutive activation of STAT5.^171^, ^174^ Therefore, STAT5 is considered a signaling hallmark of CML, and is also known to be involved in the development of other types of leukemia, such as AML and T‐cell‐derived leukemia.^172^, ^174^

#### 3.8.1 Inhibitors of STAT3/5

The development of molecules capable of inhibiting STAT3/5 activity has been the target of investigation in the last 20 years, however, given the high degree of homology in the SH2 domain between the various members of the STAT family, it has been difficult to obtain selective compounds.^173^, ^175^ Furthermore, studies show that inhibitors can only partially inhibit the transcriptional activity of these proteins.^173^, ^175^

#### 3.8.2 PROTACs against STAT3/5

**FIGURE 9:**
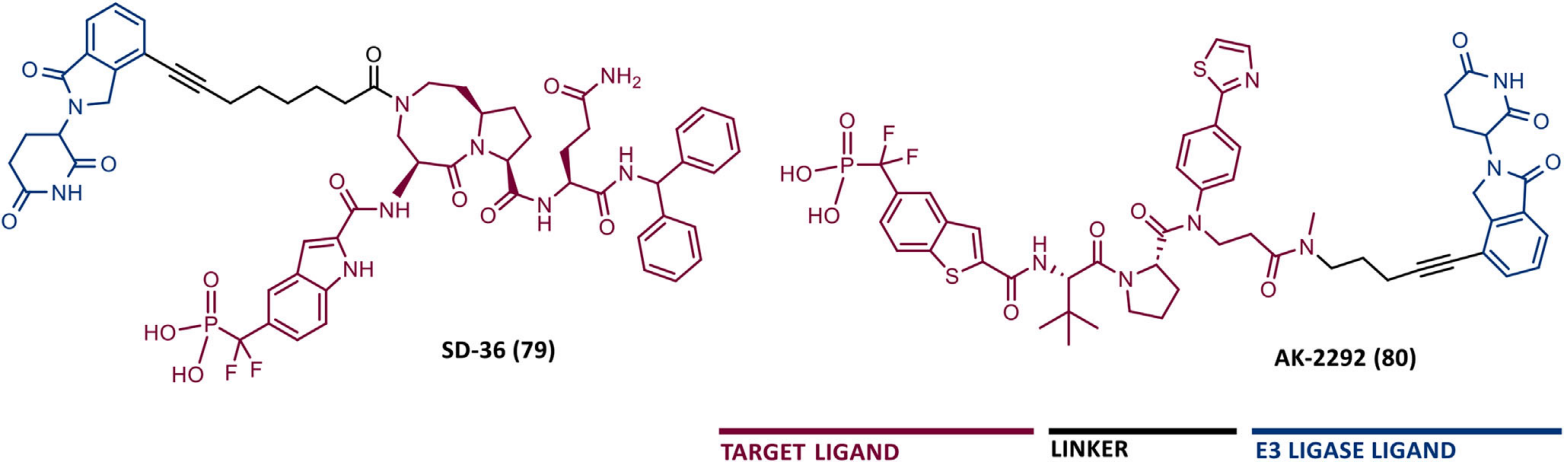
Chemical structure of PROTACs targeting STAT3/5. Analysis of the structure of PROTACs targeting the STAT3/5 proteins against leukemia, where the blue part represents the E3 ligase ligand, and the purple part represents the target ligand, joined together through a linker.

In 2019, Bai et al.^173^ developed PROTAC SD‐36 (79), capable of effectively, potently, and selectively degrading the STAT3 protein, in vitro and in vivo, in turn inhibiting the growth of leukemic cells (Figure 9).^173^, ^176^ Through a structure‐based design strategy starting from the inhibitor CJ‐887, inhibitors SI‐108 and SI‐109 are synthesized, with methyl and N‐acetyl substituents, respectively.^173^, ^176^ Both compounds are cell‐permeable and with high affinity for STAT3, capable of inhibiting its transcriptional activity (IC_50 _= 3 μM).^176^ Among the several STAT3 degraders obtained from the conjugation of compounds SI‐108 and SI‐109 to CRBN and VHL ligands, through different types of linkers, the CRBN‐based PROTAC SD‐36 stands out, which presents the SI‐109 inhibitor as TLM (Ki = 9 nM) and lenalidomide as ELM.^173^ In MOLM‐16 cells, after 4 h of treatment, SD‐36 degraded more than 90% of STAT3, demonstrating a time‐ and concentration‐dependent activity.^173^ It should be noted that at concentrations as high as 10 μM the Hook effect was not observed.^173^ Selectivity studies demonstrated that in AML cells, PROTAC SD‐36 only promoted the degradation of STAT3.^173^ In human peripheral blood mononuclear cells (PBMCs) that express all STATs, there was only a reduction in STAT3 levels, proving the selectivity of this degrader for the target.^173^ When the impact that STAT3 degradation has at the cellular level was studied, it was observed that SD‐36 at 1 μM reduces the ability of STAT3 to bind to DNA.^173^ This effect is only possible to observe with the inhibitor SI‐109 at 10 μM, in MOLM‐16 cells.^173^ Furthermore, in the same cell line, SD‐36 strongly inhibits cell growth (IC_50 _= 35 nM), being around 100 times more potent than the inhibitor SI‐109, inducing an arrest of the G1‐S transition of the cell cycle and cell apoptosis.^173^ In mouse xenograft MOLM‐16 tumors, a single intravenous dose of SD‐36 of 25 mg/kg resulted in a significant (>80%, 1 h and >95%, 6 h) and long‐lasting degradation (STAT3 levels below 50% for 4 days after a single administration) of STAT3 protein, culminating in complete tumor regression.^173^

In 2023, the same group reported a new PROTAC capable of inducing tumor regression of CML cells in vivo through the degradation of STAT5.^174^ Starting from compounds capable of binding to the SH2 domain of STAT5, the AK‐305 ligand was designed, with low micromolar binding affinities to STAT5, in which its dimethylacetamide group is a suitable tethering site for binding to lenalidomide via different types of linkers.^174^ Thus, PROTAC AK‐2292 (80) is synthesized, and when tested in the KU812 CML cell line, it was shown to degrade both STAT5A and STAT5B isoforms in a time‐dependent manner, with a DC_50_ value of 110−160 nM and a *D*_max_ of ∼95 %, by Western blot analysis.^174^ It should be noted that there was no impact on the levels of other STATs, even at concentrations as high as 0.1 mM, either in CML cells or in PBMCs, being a PROTAC highly selective for STAT5.^174^ In turn, when tested on some different CML cell lines, PROTAC AK‐2292 demonstrated high growth‐inhibitory activity (IC_50_ value around 0.2–2.2 μM), as well as inducing cell apoptosis.^174^ Studies on xenograft tumor tissues of human CML NCO2 and KU812 cell lines in mice made it possible to observe that in both, a single administration of 100 mg/Kg of PROTAC resulted in an almost complete depletion of the target protein after 6 h.^174^ Even more, the present degrader lead to tumor regression in mouse xenograft models.^174^ However, it is worth noting the need for high doses in vivo, probably due to the moderate degradation capacity, low cellular permeability, and high binding to plasma proteins of this compound.^174^

Given the involvement of STAT5 in the development of AML, PROTAC AK‐2292 was further tested in human AML cell lines.^175^ In seven AML cell lines tested, AK‐2292 was shown to degrade STAT5 with DC_50_ values of around 0.05 to 0.70 μM and *D*_max_ values of >90%.^175^ In MV4;11 xenograft tumor tissue in mice, a single dose is capable of inducing complete depletion of STAT5 after 1 h, and after 24 h it was still possible to observe a reduction in STAT5 levels greater than 70%, when compared with control levels.^175^ This degradation results in an inhibition of cell growth of around 80%.^175^

In short, PROTAC AK‐2292 reveal the potential to contribute to the treatment of cancers related to STAT5 activity, such as CML and AML.^174^, ^175^

### 3.9 Other antileukemic PROTACs

**FIGURE 10:**
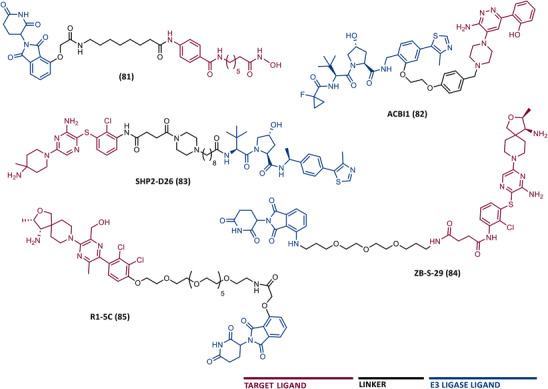
Chemical structure of other antileukemic PROTACs. Analysis of the structure of other PROTACs against leukemia, where the blue part represents the E3 ligase ligand, and the purple part represents the target ligand, joined together through a linker.

In addition to the targets discussed previously, new PROTACs have recently emerged with a view to inducing chemical knockdown of new therapeutic targets important in the development of different types of leukemia (Figure 10).

Among these new targets are the histone deacetylases (HDACs) family. HDACs are proteins involved in the regulation of genetic transcription.^177^ In 2022, Hausen's group presented a series of new CRBN‐based PROTACs targeting HDAC6, via a solid‐phase parallel synthesis approach.^178^ Among the PROTACs synthesized, PROTAC 81 stands out, which has a DC_50_ value of 3.5 nM, and a *D*_max_ of 80% when tested on AML cell lines.^178^ In MOLM‐13 cells, the induction of apoptosis was observed after treatment with the HDAC6 degrader (PROTAC 81).^178^

The induction of degradation of SMARCA2/4 subunits of PBAF chromatin remodeling complexes by PROTACs was also studied in AML cells dependent on SMARCA4 ATPase activity, by the Ciullis’ laboratory.^179^ This study reported the VHL‐based PROTAC ACBI1 (82), obtained by a two‐step synthesis process, capable of degrading SMARCA2 (DC_50 _= 6 nM) and SMARCA4 (DC_50 _= 11 nM) in MV4;11 cells.^179^ In the same cell culture, ACBI1 conferred antiproliferative activity, by inducing apoptosis, with an IC_50_ value of 28 nM.^179^

Another example of a new therapeutic target, whose degradation induced by PROTACs has been studied in leukemic cells is SHP2, which is a nonreceptor tyrosine phosphatase involved in some cancer cell signaling transductions, being associated with 4% cases of AML and in 7% cases of B‐cell ALL.^180^ The first PROTACs targeting SHP2 were presented in 2020 by Wang et al., in which the SHP099 inhibitor was connected to a VHL ligand using rigid linkers.^180^ This study resulted in the lead compound SHP2‐D26 (83), which promoted complete degradation of SHP2 in AML cells (MV4;11) with a concentration of 100 nM after 12 h, with a DC_50_ value of 2.6 nM.^180^ Compared with the SHP099 inhibitor isolated, the effect obtained by the degrader was approximately 30 times greater.^180^

In the following year, Yang et al.^181^ presented the CRBN‐based PROTAC ZB‐S‐29 (84), which degrades SHP2 with a DC_50_ value of 6 nM in MV4;11 cells, however with an effect only two times greater than that obtained with SHP099 inhibitor alone.

Recently, new CRBN‐based PROTACs targeting SHP2 were reported, in which TLM is an RMC‐4550 inhibitor, and ELM is pomalidomide. PROTAC R1‐5C (85) stands out, capable of selectively degrading the target in MV4;11, and consequently reducing their cell proliferation.^182^

## 4 ANTILEUKEMIC PROTACS IN CLINICAL TRIALS

According to the Clinicaltrials.gov database, there are more than 20 PROTACs currently in clinical trials. Among these PROTACs, at the end of 2023, six degraders were in phase I clinical trials against hematological malignancies, most of them for the treatment of B cell malignancies (Table 6).^183^ The clinical trials of BGB‐16673, NX‐5948, and NX‐2127 degraders include studies in CLL patients.^183^ However, in the clinical trials of DT2216, ABBV‐101, and AC676 degraders do not specify which the types of hematological cancers will be studied.^183^ It should be noted that most PROTACs under study aim at the forced degradation of the BTK proteins.

**TABLE 6:** PROTACs for the treatment of hematological malignancies in clinical trials.^a^

Year	Leukemia type	PROTAC	Target	E3 ligase recruited	Sponsor	Phase	NCT
2021	–	DT2216	BCL‐XL	Von Hipple‐Lindau	Dialectic Therapeutics, Inc.	I	NCT04886622
2021	CLL	NX‐2127	BTK	Cereblon	Nurix Therapeutics, Inc.	I	NCT04830137
2021	CLL	BGB‐16673	BTK	Cereblon	BeiGene	I / II	NCT05006716
2022	CLL					I	NCT05294731
2022	CLL	NX‐5948	BTK	Cereblon	Nurix Therapeutics, Inc.	I	NCT05131022
2023	–	ABBV‐101	BTK	–	AbbVie	I	NCT05753501
2023	–	AC676	BTK	–	Accutar Biotechnology, Inc.	I	NCT05780034

Abbreviation: CLL, chronic lymphocytic leukemia.

^a^ Information from the *Clinicaltrials.gov* database (April, 2024), with the keywords: “leukemia,” “hematologic cancer,” “hematologic malignancy,” “PROTAC,” and “degrader”.

As previously mentioned, PROTAC DT2216 is a specific BCL‐XL protein degrader, with high cytotoxicity against MOLT‐4 T‐ALL cells in vitro, without relevant platelet toxicity.^99^ Currently, this degrader is in phase I clinical trials. Recently, a study reported that there was no relationship between the concentrations of PROTAC DT2216, which were determined to be initially cytotoxic for the different T‐ALL cell lines, with the intracellular levels of BCL‐XL, BCL‐2, or MCL‐1 proteins.^184^ Therefore, although the DT‐2216 resistance in several T‐ALL cells is rare, the authors claim that its occurrence cannot be predicted based on measurement of BCL‐2 and MCL‐1 proteins, before and after treatment with PROTAC DT2216.^184^

## 5 POTENTIAL FUTURE TARGETS

In addition to all the therapeutic targets previously presented, there is still a huge range of proteins whose forced degradation through the use of PROTACs has not yet been tested. Among the new potential therapeutic targets, there is nucleophosmin (NPM1), a cytoplasmic localization protein frequently mutated in AML.^185^, ^186^ The design of PROTACs targeting NPM1 by recruiting a cytoplasmic E3 ligase could be advantageous as it could allow obtaining more selective compounds than current ligands. On the other hand, this is also a frequently mutated protein, and as PROTACs are less susceptible to mutations, could be another advantage.

The design of PROTACs against fusion oncoproteins, similar to what was seen in the degradation of BCR‐ABL in CML, should also be considered. Examples of these types of proteins are: AML‐specific (MLL‐fusions, PML‐RARα or AML1‐ETO) or ALL‐specific (TCF3‐PBX1 or ETV6‐RUNX1) fusion oncoproteins.^187^, ^188^, ^189^, ^190^

Furthermore, unlike SMIs that bind to the active site of targets, PROTACs do not require binding to a specific site on proteins.^191^ In other words, PROTACs only need to bind to the POI, which can be in any of its domains, and form a stable complex with the E3 ligase.^62^ In this way, proteins without an active site, and which previously could not be inhibited by conventional therapies, known as “undruggable proteins” (e.g., TFs or scaffolding proteins), can now be targeted by PROTACs.^54^ An example of a potential TF is FOXO3A (a forkhead TF) implicated in the development of AML.^192^ Furthermore, PROTACs also have the ability to promote the degradation of protein aggregates.^13^

## 6 DISCUSSION AND FUTURE PERSPECTIVES

The exponential investigation of PROTACs is the result of their revolutionary mechanism of action, in which these innovative bimolecular compounds direct a POI to the UPS, thus promoting its targeted degradation, with advantages over occupancy‐driven inhibition by SMIs (Table 7).^193^, ^194^

**TABLE 7:** Advantages and disadvantages of small molecule inhibitors (SMIs) and antileukemic PROTACs.

	Small molecule inhibitors	PROTAC
Adverse effects	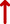	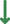
Cell permeability	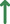	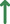
Drug design	Rational	Trial and error
Drug resistance	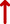	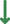
E3 ligase dependence	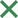	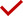
Easily be combined with other treatments	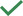	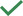
Effective period	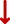	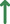
Extracellular targets	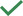	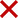
Mechanism of action (MoA)	Occupancy‐driven MoA	Event‐driven MoA
Oral administration	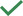	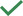
Range of clinical uses	Broad	Broad
Selectivity	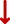	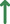
Solubility	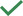	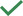
Systemic delivery	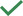	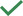
Target degradation	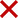	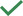
UPS dependence	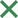	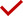
‘Undruggable’ targets	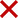	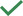

Higher (↑); lower (↓); yes (√); no (×); disadvantage (red); advantage (green).

After a general review of the molecular structure, design concepts, biological activity, and main advantages presented by PROTACs with antileukemic activity reported over the last few years, it is still important to reflect on the future challenges of these new compounds.

The overwhelming majority of antileukemic PROTACs are in preclinical development, but it is already possible to highlight several promising degraders that fulfill the purpose of degrading quickly, very selectively, with a prolonged duration of action and with potency in the order of pico‐ to nanomolar, several leukemic target proteins (respective isoforms and mutant forms) involved in the development of various types of leukemia, in vitro and in vivo. Its event‐driven mechanism—in which a PROTAC molecule can induce the degradation of several protein units (sub‐stoichiometric catalytic action)—results in reduced dose and frequency of administration, and therefore minimizes the occurrence of off‐target effects. By degrading the target, PROTACs prevent the intracellular accumulation of POI through the compensatory expression often observed in the stabilization of an inactive conformation by inhibitors. Furthermore, it is worth highlighting the greater resilience of PROTACs to the occurrence of resistance, as well as the advantages associated with their combined use with conventional reference therapies, with a significant improvement in the results observed in leukemic patients.

Any of the PROTAC components (TLM, linker, and ELM) have an impact on its performance, and it is more important to look at the combination of the three pieces than individually for each one, to increase cooperativity between them to form a stable ternary complex.^60^, ^114^, ^195^ From the analysis of the PROTACs presented previously, it is possible to conclude that the choice of TLMs has fallen on the choice of already known inhibitors, or their derivatives, and there is no complete novelty in this aspect. However, there is a huge opportunity for innovation, either through the possibility of targeting new leukemic targets (e.g., undruggable proteins), or through the incorporation of new ligands targeting new sites, other than the classic active site, of therapeutic targets.^196^ Furthermore, ligands with greater affinities do not necessarily need to be incorporated, as this does not always translate into more active degraders, as there could be a possible loss of their catalytic mechanism of action due to greater difficulty in disassembling the ternary complex.^197^ Thus, it is possible to recover the study of ligands that had previously been discarded due to low binding affinity or potency.^130^

Regarding the recruited E3 ligases, most antileukemic PROTACs incorporate ligands for the CRBN and VHL enzymes, with few examples of successful PROTACs recruiting other E3 ligases (IAP, MDM2, RNF114). However, it is necessary to consider that there is a high number of ligases available (∼600 E3 ligases), and that the choice of E3 ligase has an impact on the activity of PROTAC, both in its degradational activity and in its selectivity by impacting the interactions protein–protein (POI–E3 Ligase).^60^ To improve the safety of degraders, it would be positive to choose new selective E3 ligase ligands (selective for the enzyme, and without affecting its activity), and try to avoid what is observed with CRBN‐based PROTACs, which destabilize IKZF1/3 proteins.^198^ To reduce off‐target effects, we must also choose to recruit E3 ligases with more specificity, that is, only present at the intended site of action, as well as, evaluating the impact that PROTAC, when recruiting a certain E3 ligase, has on the general degradation of other proteins.

Regarding the choice of linker to make the connection between the two ligands, this largely relies on the incorporation of intraoxygenated chains (e.g., PEG) or alkyl chains, sometimes incorporating less flexible structures (e.g., triazole groups or aromatic rings). The study of the linker, and its characteristics (rigidity, lipophilicity, composition, length, etc.) is relevant, as it influences the physicochemical properties of PROTAC, such as solubility, metabolic stability, or cellular permeability.^198^ The linker, together with its binding site—the solvent‐exposed region of each ligand—and the linkage point, determine the geometry of the ternary complex, and consequently the activity, stability, and selectivity of PROTAC, as previously analyzed.^103^, ^114^

One of the many advantages of the antileukemic PROTACs over conventional inhibitors is their greater selectivity, which means they have less potential for off‐target effects. For example, while the inhibitor navitoclax has been shown to be equally cytotoxic to both MOLT‐4 leukemia cells and platelets, PROTAC XZ739, which targets the BCL‐XL protein, has been shown to have 100 times more selective cytotoxic activity against MOLT‐4 leukemia cells.^102^ This is an advantage because it reduces the risk of thrombocytopenia caused by the degrader, making it potentially safer than the inhibitor. Another example is the palbociclib‐based PROTACs reported by Rao et al.^149^ All of their PROTACs were much more selective, degrading only the CDK6 protein^149^. On the other hand, the respective isolated palbociclib inhibitor inhibits not only CDK6 but also CDK4.^149^ This improved selectivity of PROTACs is explained by the new protein–protein interactions that are established during the formation of the ternary complex (target–PROTAC–E3 ligase), favoring the binding of PROTAC to one target to the detriment of another. These protein–protein interactions are strongly influenced by the geometry of PROTAC, thus the linker influences both the activity and the selectivity of PROTAC.

In addition to geometry influencing PROTAC selectivity (since it influences protein–protein interactions), the spatial pattern of surface lysine residues where ubiquitin binds the target to be recognized by the E3 ligase also impacts selectivity.^103^, ^105^, ^199^ Thus, the combination of these various aspects means that, in general, PROTACs are more selective molecules for the target than inhibitors and should be studied. Furthermore, the incorporation of certain groups that allow temporospatial control of PROTACs, such as that seen in PROTAC 15, will be an added value and likely a subject for future research.^87^

Nowadays, although some information is already known about the SAR of PROTACs, there is still no rational design due to the heterogeneity of the degradation complex, which means that the development of new PROTACs is empirical, on a trial‐and‐error basis.^83^

As they are large lipophilic molecules, with high molecular weights, PROTACs do not follow traditional drug discovery rules, as the Lipinski's rule of five.^60^ These compounds have difficulty penetrating the interior of cells, which results in low intracellular concentrations, making both their study and their biological activity difficult.^116^, ^159^, ^172^ Furthermore, given their characteristics, they are difficult molecules to be administered orally.^60^, ^200^ Therefore, it is extremely important to optimize the various components of PROTAC, and subsequently evaluate their impact on both the pharmacodynamics and pharmacokinetics of the degrader.^201^ To date, there are still few antileukemic PROTACs that present in vivo studies to evaluate not only their pharmacodynamics, toxicity, but also their pharmacokinetics (absorption, distribution, metabolization, and excretion). Therefore, it is expected that these types of studies will be carried out in the future, given their relevance for assessing the potential of these degraders as future drugs for the treatment of leukemia.

## 7 CONCLUSION

Unquestionably, PROTACs have been a hot topic in the area of drug discovery and development, with a lot of information emerging day by day.

Enormous advances have been made in the development of antileukemic PROTACs, and there are already some PROTACs with the potential for the treatment of one or more types of leukemia (six in phase I/II of clinical trials), which have demonstrated that they can overcome some of the main difficulties associated with the use of SMIs, such as the occurrence of resistance. However, it should be noted that there is still a long way to go, with many aspects to be studied and clarified in more detail, as well as some challenges to overcome.

To sum up, it is possible to conclude that we are faced with extremely promising molecules, with enormous potential for scientific innovation, and which, in our opinion, will certainly prove to be an added value in the future treatment of one or more types of leukemia.

## AUTHOR CONTRIBUTIONS

*Conceptualization*: André T. S. Vicente and Jorge A. R. Salvador *Writing—original draft preparation*: André T. S. Vicente *Figures and tables preparation*: André T. S. Vicente *Writing—review*: André T. S. Vicente and Jorge A. R. Salvador *Supervision*: Jorge A. R. Salvador All authors have read and agreed to the published version of the manuscript.

## CONFLICT OF INTEREST STATEMENT

The authors declare no conflict of interest.

## ETHICS STATEMENT

Not applicable.

## Data Availability

Not applicable.
